# Inflammatory Stimuli and Fecal Microbiota Transplantation Accelerate Pancreatic Carcinogenesis in Transgenic Mice, Accompanied by Changes in the Microbiota Composition

**DOI:** 10.3390/cells14050361

**Published:** 2025-02-28

**Authors:** Agnieszka Świdnicka-Siergiejko, Jarosław Daniluk, Katarzyna Miniewska, Urszula Daniluk, Katarzyna Guzińska-Ustymowicz, Anna Pryczynicz, Milena Dąbrowska, Małgorzata Rusak, Michał Ciborowski, Andrzej Dąbrowski

**Affiliations:** 1 Department of Gastroenterology and Internal Medicine, Medical University of Bialystok, 15-276 Bialystok, Poland; jaroslaw.daniluk@umb.edu.pl (J.D.); adabrows@umb.edu.pl (A.D.); 2 Department of Medical Biochemistry, Medical University of Bialystok, 15-276 Bialystok, Poland; katarzyna.miniewska@umb.edu.pl; 3 Department of Pediatrics, Gastroenterology, Hepatology, Nutrition, Allergology and Pulmonology, Medical University of Bialystok, 15-276 Bialystok, Poland; urszula.daniluk@umb.edu.pl; 4 Department of General Pathomorphology, Medical University of Bialystok, 15-276 Bialystok, Poland; katarzyna.guzinska-ustymowicz@umb.edu.pl (K.G.-U.); anna.pryczynicz@umb.edu.pl (A.P.); 5 Department of Heamatological Diagnostics, Medical University of Bialystok, 15-276 Bialystok, Poland; mdabrows@umb.edu.pl (M.D.); malgorzata.rusak@umb.edu.pl (M.R.); 6 Metabolomics and Proteomics Laboratory, Department of Medical Biochemistry, Clinical Research Centre, Medical University of Bialystok, 15-276 Bialystok, Poland; michal.ciborowski@umb.edu.pl

**Keywords:** experimental mouse models, microbiota, pancreatic cancer

## Abstract

An association between gut microbiota and the development of pancreatic ductal adenocarcinoma (PDAC) has been previously described. To better understand the bacterial microbiota changes accompanying PDAC promotion and progression stimulated by inflammation and fecal microbiota transplantation (FMT), we investigated stool and pancreatic microbiota by 16s RNA-based metagenomic analysis in mice with inducible acinar transgenic expressions of KrasG12D, and age- and sex-matched control mice that were exposed to inflammatory stimuli and fecal microbiota obtained from mice with PDAC. Time- and inflammatory-dependent stool and pancreatic bacterial composition alterations and stool alpha microbiota diversity reduction were observed only in mice with a Kras mutation that developed advanced pancreatic changes. Stool *Actinobacteriota* abundance and pancreatic *Actinobacteriota* and *Bifidobacterium* abundances increased. In contrast, stool abundance of *Firmicutes*, *Verrucomicrobiota*, *Spirochaetota*, *Desulfobacterota*, *Butyricicoccus*, *Roseburia*, *Lachnospiraceae A2*, *Lachnospiraceae unclassified*, and *Oscillospiraceae unclassified* decreased, and pancreatic detection of *Alloprevotella* and *Oscillospiraceae uncultured* was not observed. Furthermore, FMT accelerated tumorigenesis, gradually decreased the stool alpha diversity, and changed the pancreatic and stool microbial composition in mice with a Kras mutation. Specifically, the abundance of *Actinobacteriota*, *Bifidobacterium* and *Faecalibaculum* increased, while the abundance of genera such as *Lachnospiraceace A2* and *ASF356*, *Desulfovibrionaceace* uncultured, and *Roseburia* has decreased. In conclusion, pancreatic carcinogenesis in the presence of an oncogenic Kras mutation stimulated by chronic inflammation and FMT dynamically changes the stool and pancreas microbiota. In particular, a decrease in stool microbiota diversity and abundance of bacteria known to be involved in short-fatty acids production were observed. PDAC mouse model can be used for further research on microbiota–PDAC interactions and towards more personalized and effective cancer therapies.

## 1. Introduction

Pancreatic ductal adenocarcinoma (PDAC) has a very high mortality rate, with a 5-year survival rate of only about 11% due to its late diagnosis and limited responses to therapies [1,2]. In recent years, tremendous efforts have been made to better understand how PDAC is initiated and progresses into such an aggressive and heterogeneous tumor. More than 90% of human PDACs bear Kirsten rat sarcoma virus (Kras) mutations, suggesting that this mutation may serve as an initiating point in pancreatic carcinogenesis, with other genetic mutations appearing during tumor progression [3]. However, the Kras mutation itself does not seem to be sufficient to initiate tumorigenesis, and further triggering factors that induce chronic inflammation are likely needed. Chronic pancreatitis, smoking, and obesity increase the risk of PDAC [1,3].

Recently, there has been growing interest in understanding the role of gut microbiota in PDAC development, progression, and clinical outcomes. Alterations in the composition of microbiota and bacterial abundance in the oral cavity, stool, and tumor tissue of PDAC patients compared to healthy controls have been described, along with a characteristic bacterial signature associated with the outcomes [4,5,6,7,8,9]. It was suggested that the stool microbiota of patients with PDAC exhibited significantly diminished alpha diversity compared to healthy controls [10]. However, data on alpha diversity analysis is contrasting across PDAC patient cohorts from different countries [10,11,12,13,14,15]. In addition, differences in taxa were found in patients with PDAC compared to healthy controls, such as increased phylum *Proteobacteria* (e.g., *Gammaproteobacteria*) and decreased phylum *Firmicutes* (e.g., *Faecalibacterium prausniztii*, *Roseburia intenstinalis*, and *Eubacterium rectale*) [15]. Microbiome modulation appears to be a promising strategy for the prevention and current treatment options of PDAC, despite the lack of sufficient evidence-based data. Most published studies have investigated the microbiota in patients with advanced PDAC, but our knowledge about the role of microbiota in the initiation and progression of PDAC is limited [11,12,13,14,15,16,17,18,19]. It was found that *Porphyromonas gingivalis*, an oral pathogen, was able to accelerate the development of precancerous lesions—pancreatic intraepithelial neoplasia (PanIN) in a mouse model by enhancing transforming growth factor β signaling [20]. On the other hand, the tumor may create a microenvironment that promotes microbiota dysbiosis, thereby modulating the PDAC progression [5,6,7,8,21]. Existing data suggest that microbiota can also modulate the response to PDAC therapy directly or indirectly via the production of microbiota-derived metabolites [18,19]. The transplantation of fecal microbiota (FMT) of PDAC patients into mice showed its capacity to modulate tumor progression. Tumor growth was decreased in mice with FMT from long-term survival PDAC patients compared to mice with FMT from short-term survival patients [21]. Mouse genetic models of PDAC can be used to test the factors and pathways influencing pancreatic carcinogenesis, as well as novel therapies, including microbiome-directed therapies. However, data on the microbiota composition in experimental mouse models of PDAC are still limited [11,16,17,20,21,22].

The causal relationship between microbiota, chronic inflammation, the initiation and progression of pancreatic carcinogenesis, and their underlying mechanisms remain to be explored further.

The aim of this study was to characterize the alterations in the stool and pancreatic bacterial microbiota community in order to identify microbiota signatures associated with the promotion and progression of pancreatic carcinogenesis in the presence of oncogenic Kras mutation and stimulated by inflammation. In addition, we have investigated the effect of FMT on PDAC development and changes in microbiota composition after FMT in relation to genetic predisposition.

To analyze how the presence of an oncogenic predisposition to PDAC and stimuli such as inflammation and FMT modulate the bacterial microbiota, we used a genetically engineered inducible mouse model with pancreas-specific expression of oncogenic Kras G12D, which has been shown to fully recapitulate the pancreatic carcinogenesis pathway leading through chronic inflammation and PanIN lesions to PDAC [23,24,25].

## 2. Materials and Methods

### 2.1. Study Objects and Protocol

We used KrasG12D mice expressing a conditional knock-in mutant KrasGI2D. A full-length elastase (Ela) gene promoter was used to drive the expression of tamoxifen-regulated CreERT specifically in adult pancreatic acinar cells in mice (Ela-CreERT) as described previously [18,19,20]. KrasG12D mice were then bred with Ela-CreERT (Cre) mice to generate Kras/Ela-CreERT (Kras/Cre) double transgenic mice. The mice were given tamoxifen orally for 3 days to initiate oncogenic Kras expression in acinar cells starting at the age of 40 days. As previously shown, this transgenic animal model fully replicates a human PDAC phenotype when exposed to inflammatory stimuli. The presence of Ela-CreERT does not affect the phenotype of the animals. Therefore, Cre mice were used as controls [23,24,25]. All experiments were carried out in accordance with the EU Directive 2010/63/EU and regulations of the Center of Experimental Medicine, Medical University of Bialystok, and approved by the Local Ethical Committee for Animal Experiments (Olsztyn, Poland).

### 2.2. Process of Pancreatic Carcinogenesis

Thirty-day-old Kras/Cre mice and Cre control mice were subjected to a 12 h dark/light cycle at 22 °C, with access to water and standard rodent chow ad libitum. For experimental analyses, sex- and age-matched animals were used. To accelerate the development of PanIN lesions and PDAC, animals were subjected to a series of cerulein injections to induce pancreatitis. Briefly, 40–50 days old Kras/Cre mice (N = 30) and Cre control mice (N = 30) were administered cerulein (50 μg/kg dissolved in 100 μL of sterile saline with 0.1% bovine serum albumin) via hourly intraperitoneal injections over 5 h on the first day, followed by one injection per day for 4 consecutive days. Two weeks after the initiation of treatment, the entire course of cerulein injections was repeated. Animals were then sacrificed by a cardiac puncture at two specified time points after the indicated treatments: 1/ 30 days (Kras/Cre mice, n = 15, and Cre mice, n = 15) and 2/ 120 days (Kras/Cre mice, n = 15, and Cre mice, n = 15) after cerulein injections. Based on our previous experiments and data from the literature, Kras/Cre mice at the age of 2 months begin to develop PanIN lesions (PanIN1) that become more numerous and dysplastic (PanIN2/3) with the mice age. We predicted that Kras/Cre mice would develop chronic pancreatitis, PanIN, and more advanced pancreatic lesions (PDAC/PanIN3) 30 days and 120 days after cerulein injections, accordingly. The Cre mice do not show features of pancreatic carcinogenesis after inflammatory stimulation [23,24,25]. Pancreas samples from all animals were removed, washed with PBS, placed in 10% formaldehyde, embedded in paraffin and sectioned onto slides, and stained with hematoxylin and eosin. All slides were assessed by a pathologist for the presence of PanIN lesions and PDAC.

### 2.3. Fecal Microbiota Transplantation

The fecal microbiota transplantation was performed according to the principles of others, with minor modifications [21,26,27]. In brief, fecal pellets from Kras/Cre mice that developed PDAC after cerulein treatment were collected and pooled together, weighted, and then placed in 0.25–1.0 mL of sterile transfer buffer. The final volume was adjusted to give 120 mg of feces per ml. The fecal pellets were then gently homogenized and centrifuged at 800× *g* for 2 min, and the supernatant was collected and diluted (1:3) in a sterile transfer buffer. Prior to fecal transplantation, the endogenous gut microbiota of Kras/Cre mice (n = 20) and control Cre mice (n = 20) aged 40–50 days was depleted with a solution containing streptomycin (5 mg/mL, Streptomycin sulfate, 1 g) and clindamycin (0.1 mg/mL, Klimicin, 300 mg/2 mL) added to the sterile drinking water of mice ad libitum for 14 days (2 changes of solutions and bottles per week). After treatment with oral antibiotics, the mice were temporarily housed on raised wire flooring to prevent coprophagy. FMT was initiated 24 h after stopping the antibiotics by placing 100 μL of donor fecal supernatant directly into the oral cavity of the recipient mice (Kras/Cre mice, n = 10 and Cre mice, n = 10). In the sham treatment group, animals (Kras/Cre mice, n = 10 and Cre mice, n = 10) received only the transfer buffer. Each group of mice received transplants (FMT or sham) 3 times in the first week and then once a week until the end of the experiment. All mice were inspected frequently and euthanized in cases of cancer suspicion or general condition deterioration (age death range of Kras/Cre mice and Cre mice: 110–130 days after the initiation of FMT/sham treatments). Stool samples were collected before antibiotics and FMT/sham treatments, during FMT/sham treatments, and after FMT/sham treatments. These samples were stored for further examination. Pancreas samples were collected at the end of the experiment after sham and FMT treatments and stored for further examinations. A postmortem examination with histological analysis of pancreatic tissues was performed as described above.

### 2.4. Sample Processing and DNA Extraction for Microbiota Analysis

After resection, pancreatic samples were immediately placed in a sterile vial on dry ice and then transferred to storage at −80 °C. Stool collection, storage, stabilization, and purification of DNA were performed using the PSP Spin Stool System (Invitek Molecular GmbH, Berlin, Germany) according to the manufacturer’s instructions. The protocol for isolating DNA from stool homogenate from difficult-to-lyse bacteria was performed as follows: sample homogenization and pre-lysis, removal of PCR inhibitors, second sample cleanup, proteinase K digestion, binding of the DNA, washing steps, ethanol removal, and DNA elution. The isolation of bacterial DNA from pancreatic tissue was conducted using the Genomic Mini AX Bacteria + (mod.6) kit (A&A Biotechnology, Gdynia, Poland) according to the manufacturer’s instructions. All DNA samples that met the quality requirements necessary for sequencing analysis, as recommended by the Genomed institution (Genomed S.A., Warsaw, Poland), were further tested.

### 2.5. 16S RNA Sequencing and Bioinformatic Analysis

The 16S rRNA gene sequencing was performed by Genomed S.A., Warsaw, Poland. In brief, metagenomic analysis of bacterial and archaeal populations was conducted based on the hypervariable V3–V4 region of the 16S rRNA gene. Specific primer sequences 341F and 785R were used to amplify the selected region and prepare the library for 16S analysis. PCR was performed using the Q5 Hot Start High-Fidelity 2× Master Mix (New Englands Biolabs, MA, Ipswich, USA), with reaction conditions following the manufacturer’s recommendations. Sequencing was performed on a MiSeq device (Illumina, Inc., San Diego, CA, USA), using paired-end (PE) technology, 2 × 300 nt, with the v3 Illumina kit (Illumina, Inc., San Diego, CA, USA). Automatic preliminary data analysis was performed on the MiSeq sequencer using MiSeq Reporter (MSR, version 2.6.2.3) software. The analysis consisted of two stages: 1. automatic demultiplexing of samples and 2. generation of fastq files containing raw reads. Bioinformatic analysis ensuring the classification of reads to the species level was carried out using the QIIME 2 software (version 2020.6) package based on the 138 Silva reference sequence database. The DADA2 package (version 2020.6.0) was also employed, which allowed for the identification of sequences of biological origin from those newly created in the sequencing process. This package was also used to extract unique sequences of biological origin, known as ASV sequence (amplicon sequence variant). The details of the analysis and references are presented in Supplementary Material S1. Additional extended bioinformatic analysis was carried out using the R program (version 3.6.3) and the phyloseq (version 1.30.0) and vegan (version 2.5-7) packages.

### 2.6. Statistical Analysis

The statistical analysis was performed using R language and environment software (version 4.3.2, https://www.R-project.org/, accessed on 14 August 2024), using different R packages (tidyverse (2.0.0), dunn.test (1.3.6)). The differences in bacteria abundances between groups were calculated using the Kruskal–Wallis test with the Dunn post-hoc test and the U Mann–Whitney test (for three or more groups and two groups, respectively). Plots were generated using the ggplot2 (3.5.1) package. Principal coordinate analysis (PCoA) and visualization were performed using vegan (2.6-6.1), ape (5.8), and ggforce (0.4.2) packages.

## 3. Results

### 3.1. Inflammation in the Presence of Oncogenic Kras Mutation Promotes Pancreatic Carcinogenesis

We have used an inducible mouse model with pancreas-specific expression of the Kras mutation, in which pancreatic carcinogenesis was accelerated via cerulein-induced inflammation [23,24,25]. For the purpose of this study, mice with Kras mutation (Kras/Cre) and without Kras mutations used as controls (Cre mice) were sacrificed 30 days and 120 days after cerulein injections (CER) and saline injections (Sal). We used this model for further analyses of changes in the stool and pancreatic bacterial microbiota during the progression of inflammation-induced pancreatic carcinogenesis.

As expected, we did not observe advanced pancreatic changes (PanIN3/PDAC) in control Cre mice with saline and cerulein injections. In contrast, advanced pancreatic changes were observed in 9% of Kras/Cre mice 30 days after saline injections and in 62.5% of Kras/Cre mice 30 days after cerulein injections. Moreover, advanced pancreatic changes were found in 68.5% of Kras/Cre mice 120 days after saline injections and in almost all (95%) of Kras/Cre mice 120 days after cerulein injections (Table S1, Figure S1). These data indicate that chronic inflammation caused by external factors in the presence of a Kras mutation stimulates the promotion and progression of pancreatic carcinogenesis.

### 3.2. Characteristics of the Bacterial Community at the Phyla Level in Inflammation-Induced Pancreatic Carcinogenesis

**Figure 1:**
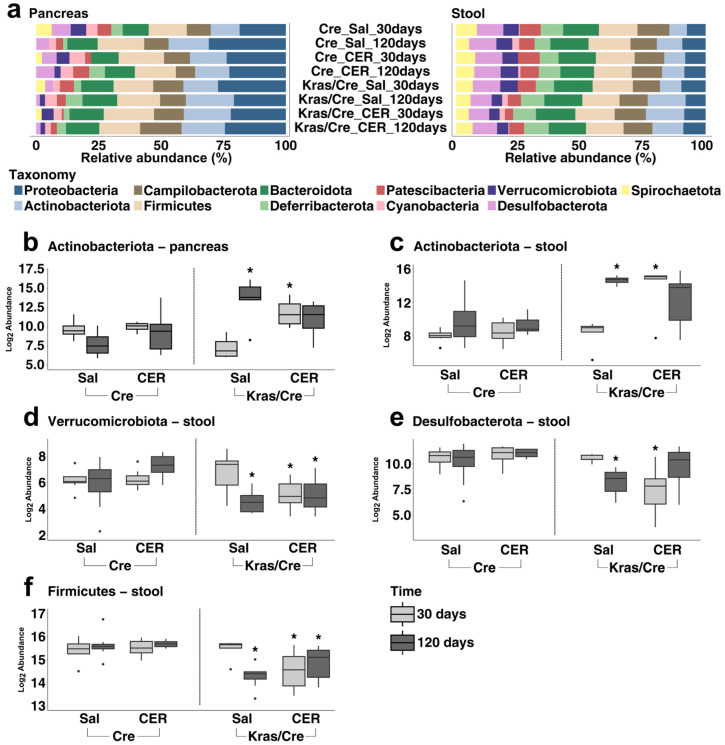
Abundance of bacterial phyla in inflammation-induced pancreatic carcinogenesis. Relative abundance (%) of most common phyla in pancreas and stool samples (**a**). The differences between Kras/Cre mice and Cre mice 30 days and 120 days after saline and cerulein injections in the abundance of *Actinobacteriota* in pancreas samples (**b**), *Actinobacteriota* in stool (**c**), *Verrucomicrobiota* in stool (**d**), *Desulfobacterota* in stool (**e**), *Firmicutes* in stool (**f**). The median abundance (%) is indicated by a black line. The box represents the interquartile range. The whiskers extend to the upper adjacent value and the lower adjacent value and dots represent outliers. Statistically significant differences (*p* value < 0.05) between mice are marked * (for details see Table S4). Sal—saline, CER—cerulein, Kras/Cre—mice with Kras mutation, Cre—mice without Kras mutation.

To characterize the bacterial microbiota community, we extracted DNA from stool and pancreas samples of all experimental mice, followed by sequencing and bioinformatics analyses. First, we analyzed the bacterial composition and abundance at the phyla level separately for all tested pancreas samples (Figure 1a, Table S2) and stool samples (Figure 1a, Table S3). In general, in the pancreas samples, the highest abundance with 100% prevalence was observed for *Proteobacteria* followed by *Firmicutes*, and then *Actinobacteriota* (Figure 1a, Table S2). In contrast, the highest abundance with 100% prevalence in the stool samples was found for *Firmicutes* followed by *Bacteroidota*, *Campilobacterota*, *Actinobacteriota*, and *Desulfobacterota* (Figure 1a, Table S3).

Next, to explore time-dependent alterations in the microbiota at the phyla level in the course of inflammation-induced pancreatic carcinogenesis, we investigated changes in bacterial abundance in Kras/Cre mice and Cre mice 30 days and 120 days after cerulein injections as well as in age and sex-matched Kras/Cre mice and Cre mice that received saline injections. Analyses were carried out separately in pancreatic and stool samples as described below.

#### 3.2.1. Pancreatic Bacterial Phyla Abundance Change in the Course of Inflammation-Induced Carcinogenesis in Mice Genetically Predisposed to PDAC

First, we analyzed if the pancreatic bacterial phyla abundance had changed after the induction and progression of carcinogenesis induced by inflammatory stimuli. We found that in mice with Kras mutation 120 days after saline injections, as well as 30 days and 120 days after cerulein injections, there was an increase in *Actinobacteriota* abundance in the pancreas samples. The Kras/Cre mice 30 days after saline injections had the lowest abundance of *Actinobacteriota* (Figure 1b). However, we did not find significant differences in the abundance of all phyla in Cre mice after both saline and cerulein injections. Interestingly, *Spirochaetota* was detected in both Kras/Cre and Cre mice 30 days after saline and cerulein injections, but it was not found 120 days after saline and cerulein injections (Table S2). This indicates that changes in pancreatic abundance of phyla can occur in a time- and inflammatory-dependent manner only in mice with oncogenic Kras mutation. Moreover, the pancreatic bacterial phyla do not change after inflammatory stimulation in the absence of an oncogenic Kras mutation.

It has been reported that human PDAC tumors harbor different microbiomes than healthy pancreatic tissue. Indeed, in our study, we found differences in bacterial phyla abundance between mice that developed PanIN/PDAC and mice that did not develop advanced pancreatic changes. As we mentioned above, mice without a Kras mutation (Cre mice) did not develop PanIN/PDAC, and significant alterations in bacterial phyla in the pancreatic samples were not observed. In contrast, our data indicate that the presence of advanced pancreatic changes is accompanied by higher pancreatic *Actinobacteriota* abundance.

#### 3.2.2. Stool Bacterial Phyla Abundance Significantly Changes in the Course of Inflammation-Induced Carcinogenesis in Mice Genetically Predisposed to PDAC

Gut dysbiosis was found in human PDAC and spontaneous KPC mouse models compared to the healthy pancreas; however, there is limited knowledge as to how the gut microbiota changes with the initiation and progression of pancreatic carcinogenesis [10,11,12,13,14,15,16,17,18,19,21]. Therefore, we investigated the alterations in the composition and abundance of stool bacterial phyla that occur after inflammatory stimulation in relation to the presence of a Kras mutation. First, we did not find significant differences in the stool phyla abundance after both saline and cerulein injections in mice without a Kras mutation. In contrast, there were changes in bacterial phyla in stool samples in mice with a Kras mutation (Figure 1c–f, Table S3). Specifically, the abundance of *Actinobacteriota* was significantly higher in Kras/Cre mice 120 days after saline injection and 30 days after cerulein injections compared to the Kras/Cre mice 30 days after saline injections and Cre mice. Furthermore, the abundance of *Actinobacteriota* was higher in Kras/Cre mice compared to Cre mice (Figure 1c). In contrast, we have observed a decrease in the abundance of *Verrucomicrobiota* (Figure 1d), *Desulfobacterota* (Figure 1e), and *Firmicutes* (Figure 1f) in Kras/Cre mice 30 days and 120 days after cerulein injections and 120 days after saline injections. These data indicate that the alterations in stool bacterial phyla abundance occur in a time- and inflammatory-dependent manner only in the presence of an oncogenic Kras mutation. In contrast, the stool bacterial phyla do not change after inflammatory stimulation in the absence of an oncogenic Kras mutation.

These observations and mice histopathology show that the stool phyla composition is altered in the presence of advanced pancreatic changes and the initiation and progression of pancreatic carcinogenesis is accompanied not only by a significant increase in stool *Actinobacteriota* abundance as it was observed in pancreas samples, but also by a significant decrease in *Firmicutes*, *Verrucomicrobiota,* and *Desulfobacterota* abundances.

### 3.3. Characteristics of the Bacterial Community at the Genera Level in Inflammation-Induced Pancreatic Carcinogenesis

**Figure 2:**
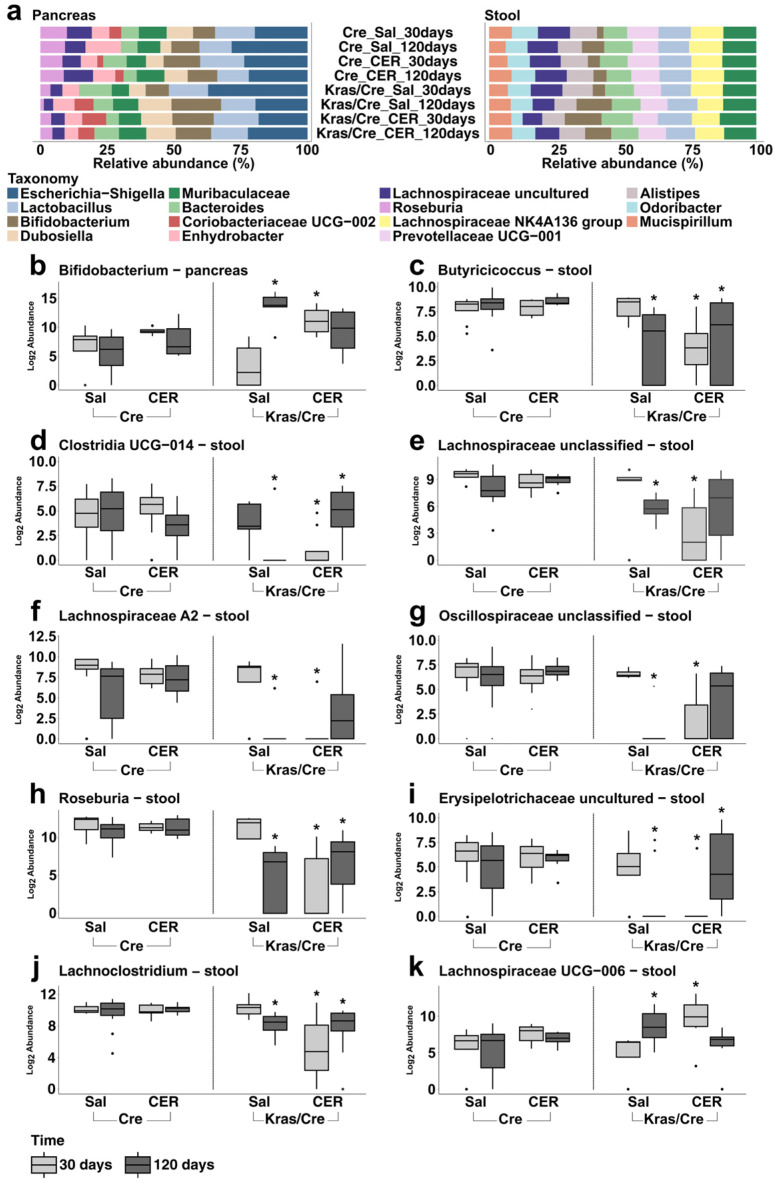
Abundance of bacterial genera in inflammation-induced pancreatic carcinogenesis. Relative abundance of most common phyla in pancreas and stool samples (**a**). The differences between Kras/Cre mice and Cre mice 30 days and 120 days after saline and cerulein injections in abundance of *Bifidobacterium* in pancreas samples (**b**), *Butyricicoccus* in stool (**c**), *Clostridia UCG-014* in stool (**d**), *Lachnospiraceae unclassified* in stool (**e**), *Lachnospiraceae A2* in stool (**f**), *Oscillospiraceae unclassified* in stool (**g**), *Roseburia* in stool (**h**), *Erysipelotrichaceae uncultured* in stool (**i**), *Lachnoclostridium* in stool (**j**) and *Lachnospiraceae UCG-006* in stool (**k**). The median abundance (%) is indicated by a black line. The box represents the interquartile range. The whiskers extend to the upper adjacent value and the lower adjacent value and dots represent outliers. Statistically significant differences (*p* value < 0.05) between mice are marked * (for details: see Table S7). Sal—saline, CER—cerulein, Kras/Cre—mice with Kras mutation, Cre—mice without Kras mutation.

To extend the observations on microbiota alterations at the phyla level, we investigated the bacterial composition and abundance at the genera level in pancreas and stool samples. The most common bacterial genera observed in pancreas and stool samples are visually presented in Figure 2a. In general, *Escherichia-Shigella* was the most abundant genera in pancreas samples (prevalence 100%) followed by other genera such as *Lactobacillus*, *Bifidobacterium*, *Dubosiella,* and *Muribaculaceae* (Figure 2a, Table S5). In contrast, *Muribaculaceae* (prevalence 100%) was the most abundant followed by genera such as *Lactobacillus*, *Lachnospiraceae* (*uncultured* and *NK4A136 group*), *Bacteroides*, and *Bifidobacterium* in stool samples (Figure 2a, Table S5).

Next, we compared the genera abundance in Kras/Cre mice and Cre mice 30 days and 120 days after cerulein injections as well as in age and sex-matched Kras/Cre mice and Cre mice that received saline injection in relation to the mice histopathology to explore the abundance of genera in the stool and pancreas samples in the course of inflammation-induced pancreatic carcinogenesis and to identify genera associated with PDAC.

#### 3.3.1. Pancreatic Bacterial Genera Composition and Abundance Dynamically Change in the Course of Inflammation-Induced Carcinogenesis in Mice Genetically Predisposed to PDAC

First, we assessed whether there were changes in bacterial genera abundance in pancreas samples in all experimental mice. We did not observe significant differences in the abundance of bacteria in mice without Kras mutation (Cre mice). Moreover, the abundance of bacterial genera in Kras/Cre mice 30 days after saline injections was not significantly different from Cre mice. However, we found that Kras/Cre mice 120 days after saline injections and after cerulein injections had increased *Bifidobacterium* abundance compared to Kras/Cre mice 30 days after saline injections (Figure 2b).

Furthermore, the analysis of taxonomic profiles in pancreatic samples revealed specific bacteria that were not detected in Kras/Cre mice 120 days after saline injections or, 30 and 120 days after cerulein injections. These included *Alloprevotella* and *Oscillospiraceae uncultured* which were found to be present in Kras/Cre mice 30 days after saline injections and in Cre mice. Moreover, we did not find *Colidextribacter*, *Oscillibacter*, and *Incertae Sedis* in Kras/Cre mice 30 and 120 days after cerulein injections as opposed to the other groups of mice (Table S8).

Taken together, the above data and histopathology results confirm that the promotion and progression of pancreatic carcinogenesis are associated with changes in the pancreatic composition of bacterial genera. Specifically, the development of PanIN3/PDAC was accompanied by a significant increase in the pancreatic abundance of *Bifidobacterium*. It may suggest that pancreatic carcinogenesis related to the presence of a Kras mutation as well as cerulein stimulation is accompanied by the eradication of some bacterial genera in pancreatic tissue.

#### 3.3.2. Stool Bacterial Genera Abundance Dynamically Change in the Course of Inflammation-Induced Carcinogenesis in Mice Genetically Predisposed to PDAC

Next, we assessed if specific genera significantly changed in the stool of mice with a Kras mutation and induced by inflammatory stimulation progression of the disease. First, in Kras/Cre mice 120 days after saline injections and 30 days and 120 days after cerulein injections we noticed that the abundance of several genera was different compared to Kras/Cre mice 30 days after saline injections and control Cre mice. In addition, there were no differences in the abundance of these genera between Cre mice and Kras/Cre mice 30 days after saline injections (Figure 2c–k). Specifically, the development of advanced pancreatic changes was accompanied by a decrease in the abundance of *Butyricicoccus*, *Clostridia UCG-014*, *Lachnospiraceae unclassified*, *Lachnospiraceae A2*, *Oscillospiraceae unclassified*, *Roseburia, Erysipelotrichaceae uncultured,* and *Lachnoclostridium* as well as an increase in *Lachnospiraceae UCG-006*. However, the decrease in genera abundance was particularly noticeable in Kras/Cre mice 120 days after saline and 30 days after cerulein injections, but not in Kras/Cre mice 120 days after cerulein injections. Taken together, these data confirm that pancreatic carcinogenesis is associated with the alteration of gut microbiota composition at the genera level. Specifically, the reduction in the abundance of several bacterial genera was found only in mice with oncogenic Kras mutation that developed pancreatic tumors.

### 3.4. The Microbiota Alpha and Beta Diversities in Inflammation-Induced Pancreatic Carcinogenesis

To extend our understanding of the role of microbiome diversity in inflammation-induced pancreatic carcinogenesis, we investigated the alpha and beta diversities of pancreas and stool microbiota separately. In general, alpha diversity is a measure of microbiome diversity within a community, while beta diversity is a measure of the similarity or dissimilarity of two communities.

**Figure 3:**
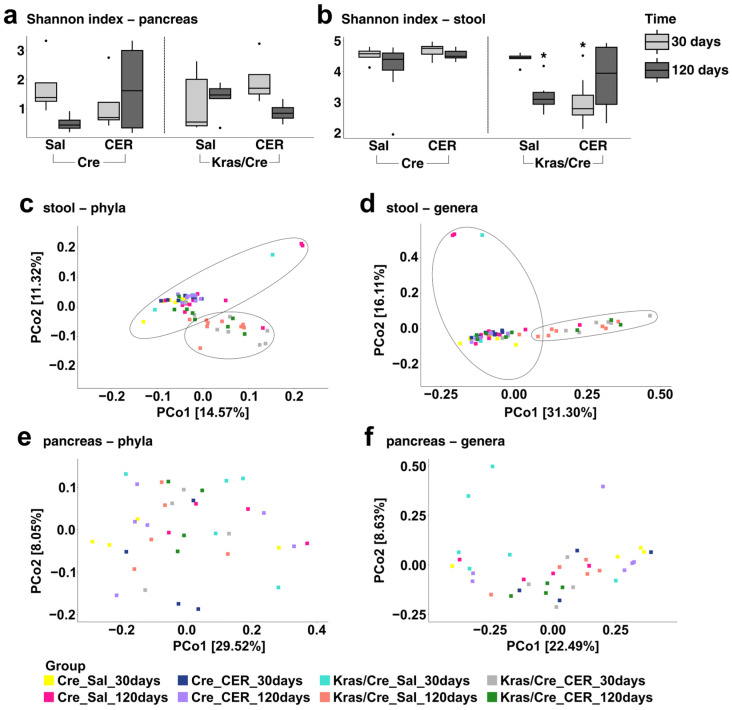
The microbiota diversity in pancreas and stool samples in inflammation-induced carcinogenesis. Shannon index—pancreas (**a**), Shannon index—stool (**b**), Principal coordinates analysis (PCoA)—stool phyla (**c**), Principal coordinates analysis (PCoA)—stool genera (**d**), Principal coordinates analysis (PCoA)—pancreas phyla (**e**), Principal coordinates analysis (PCoA)—pancreas genera (**f**). (**a**,**b**): The median alpha diversity (Shannon index) is indicated by a black line. The box represents the interquartile range. The whiskers extend to the upper adjacent value and the lower adjacent value and dots represent outliers. Statistically significant differences (*p* value < 0.05) between mice are marked * (for details see Table S11). Sal—saline, CER—cerulein, Kras/Cre—mice with Kras mutation, Cre—mice without Kras mutation.

We measured the alpha diversity using Shannon and Simpson diversity indexes that incorporate both the relative abundance and total count of distinct bacteria (Figure 3a,b, Tables S9 and S10A,B). In the alpha diversity analysis, a significant decrease in microbiota diversity in the stool was observed in Kras/Cre mice 120 days after saline injections and 30 days after cerulein injections in comparison to Kras/Cre mice 30 days after saline injections and Cre mice (Figure 3b, Table S10B). In addition, there were no differences in bacterial diversity among Kras/Cre mice 120 days after saline injections, 30 days after cerulein injections, and 120 days after cerulein injections. There were no significant differences in the stool alpha diversity in Cre mice after both saline and cerulein injections. Our findings suggest the decrease in the stool alpha microbiota diversity occurs only in mice with a Kras mutation with the promotion and progression of pancreatic carcinogenesis. A similar analysis of bacterial alpha diversity was performed in pancreas samples. However, we did not note significant differences in pancreatic alpha diversity of the microbial composition between all tested groups (Figure 3a, Table S10A).

To further elucidate the dependencies between groups we performed a beta diversity analysis for stool samples using the Bray–Curtis dissimilarity distance. This compares the diversity between communities by measuring their distance and takes into account both the number of taxa in the sample and their phylogenetic relationship. For better visualization of the results, we used the PCoA plot that indicates two clusters on both the phylum and genus levels (one for mice without advanced pancreatic changes [Cre mice, Kras/Cre mice 30 days after saline injections] and one for mice with advanced pancreatic changes [Kras/Cre mice 120 days after saline injections and 30 days and 120 days after cerulein injections]) (Figure 3c,d). The clusters were generated based on a k-means method and the differences between them were significant (R^2^ = 0.33, *p* = 0.001 for phylum; R^2^ = 0.37, *p* = 0.001 for genus). The observation that the Kras/Cre mice 120 days after saline injections cluster together with the Kras/Cre mice 30 and 120 days after cerulein injections suggests that the composition of the gut microbiota in mice with advanced pancreatic changes is distinguishable from mice without these changes. In addition, it seems that changes in the stool microbiota community progress with carcinogenesis more rapidly due to inflammation. We performed analogous analyses for pancreatic tissue samples. However, there was no clear clustering on PCoA plots indicating no differences between the analyzed groups (Figure 3e,f). We performed analogous analyses for pancreatic tissue samples as well. There was no clear clustering on PCoA plots indicating no differences between the analyzed groups (Figure 3e,f).

### 3.5. Fecal Microbiota Transplantation Influences Pancreatic Carcinogenesis in Mice Genetically Predisposed to PDAC

The more pronounced changes in the bacterial microbiota observed in the stool samples compared to pancreas samples in mice with a Kras mutation that developed advanced pancreatic changes in the above experiments may suggest that gut dysbiosis is not only a result of PDAC development but also a driving force in the initiation and progression of pancreatic carcinogenesis. Therefore, we investigated whether the application of fecal microbiota from mice with pancreatic cancer will influence pancreatic carcinogenesis in Kras/Cre mice and control Cre mice.

We did not observe histological abnormalities in Cre mice after FMT or sham treatments. In contrast, all Kras/Cre mice after FMT had macroscopic tumors and PDAC was confirmed histologically in 62.5% of mice and PanIN lesions in 37.5% of mice. PDAC and PanIN lesions were both diagnosed in 40% of Kras/Cre mice after sham treatment (Table S1, Figure S2). This may suggest that gut dysbiosis due to PDAC can promote and accelerate the development of advanced pancreatic changes in the presence of genetic predisposition.

### 3.6. Characteristics of the Bacterial Community at the Phyla Level After Fecal Microbiota Transplantation

**Figure 4:**
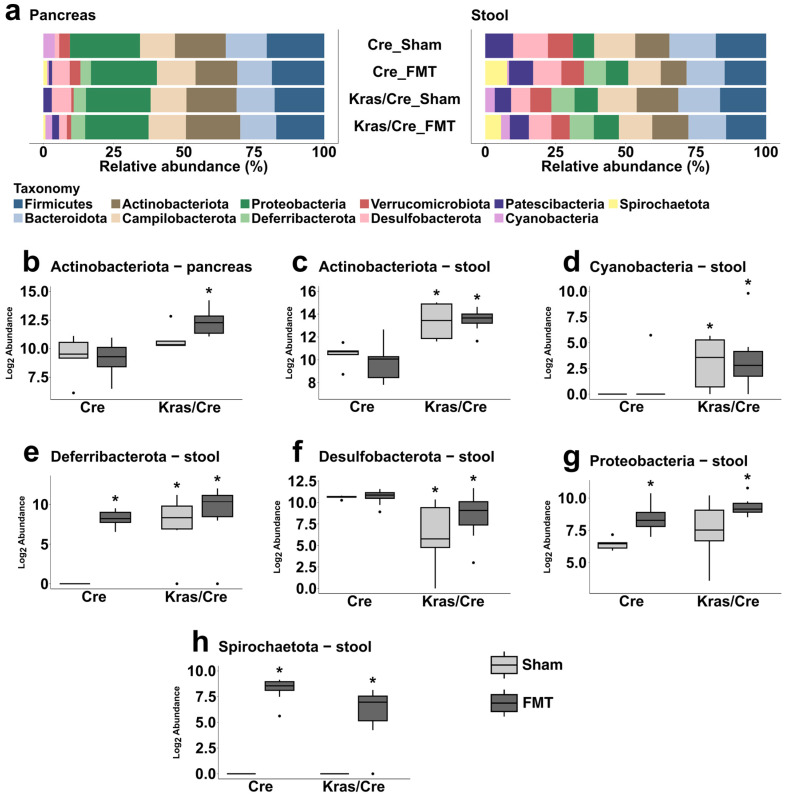
Abundance of bacterial phyla after fecal microbiota transplantation associated pancreatic carcinogenesis. Relative abundance of the most common phyla in pancreas and stool samples in Kras/Cre mice and Cre mice after FMT and sham treatments (**a**). The differences between tested mice in abundance of *Actinobacteriota* in pancreas sample (**b**), *Actinobacteriota* in stool (**c**), *Cyanobacteria* in stool (**d**), *Deferribacterota* in stool (**e**), *Desulfobacterota* in stool (**f**), *Proteobacteria* in stool (**g**), and *Spirochaetota* in stool (**h**). The median abundance (%) is indicated by a black line. The box represents the interquartile range. The whiskers extend to the upper adjacent value and the lower adjacent value and dots represent outliers. Statistically significant differences (*p* value < 0.05) between mice are marked * (for details see Table S14). Kras/Cre—mice with Kras mutation, Cre—mice without Kras mutation, FMT—fecal microbiota transplantation.

The microbiota composition after FMT and sham treatments was assessed separately in stool samples and pancreas samples. In general, the most common phylum detected in all tested pancreas samples was *Proteobacteria.* The most common phyla in stool samples was *Firmicutes* (Figure 4a, Tables S12 and S13).

#### 3.6.1. Fecal Microbiota Transplantation Alters the Pancreatic Bacterial Phyla Abundance in Mice Genetically Predisposed to PDAC

We posed the question if the development of advanced pancreatic changes observed after FMT from PDAC mice is associated with changes in the taxonomic composition and abundance of pancreatic bacterial phyla. Therefore, we investigated if the abundance of bacterial phyla in pancreatic samples after FMT differs between mice with a Kras mutation and those without it.

We found that among all phyla only the abundance of *Actinobacteriota* changed in pancreatic samples and was higher in Kras/Cre mice after FMT than in Cre mice after FMT, but it was not significantly different compared to Kras/Cre mice after sham treatment (Figure 4b). This indicates that in mice with a Kras mutation, the alteration in the pancreatic *Actinobacteriota* abundance occurs in relation to the development of advanced pancreatic changes independently of FMT or sham treatments.

Interestingly, FMT resulted in the identification of *Spirochaetota* in pancreas samples of both Cre and Kras/Cre mice but was not detected in the sham arms. In general, we did not find significant differences between the pancreatic microbiota of Kras/Cre mice and Cre mice in the sham arms (Table S12). It seems that FMT was the main source of *Spirochaetota* in pancreas samples.

#### 3.6.2. Fecal Microbiota Transplantation Alters the Stool Bacterial Phyla Abundance in Mice Genetically Predisposed to PDAC

In contrast to pancreas samples, we observed several differences between study groups in the abundance of phyla such as *Actinobacteriota, Cyanobacteria*, *Deferribacterota*, *Desulfobacterota*, *Proteobacteria,* and *Spirochaetota* in stool samples (Figure 4c–h, Table S13). Specifically, *Actinobacteriota* and *Cyanobacteria* abundances significantly increased while *Desulfobacterota* abundance decreased in Kras/Cre mice after both FMT and sham treatments compared to Cre mice (Figure 4c,d). In addition, there was a significant increase in the *Deferribacterota* abundance in Kras/Cre mice after both FMT and sham treatments as well as in Cre mice after FMT compared to Cre mice after sham treatments (Figure 4e, Table S13).

We found that in Kras/Cre mice only two phyla, *Proteobacteria* and *Spirochaetota,* increased with FMT compared to sham treatments, but a similar observation was made for Cre mice. This may suggest that FMT was the main source of these two phyla in mice stool (Figure 4g,h).

Taken together, these data indicate that FMT from PDAC mice does not significantly change the stool abundance of most phyla in the absence of an oncogenic Kras mutation. Indeed, more pronounced alterations in stool phyla occur only in mice with a Kras mutation that developed tumors independently of treatments.

### 3.7. Characteristics of the Bacterial Community at the Genera Level After Fecal Microbiota Transplantation

**Figure 5:**
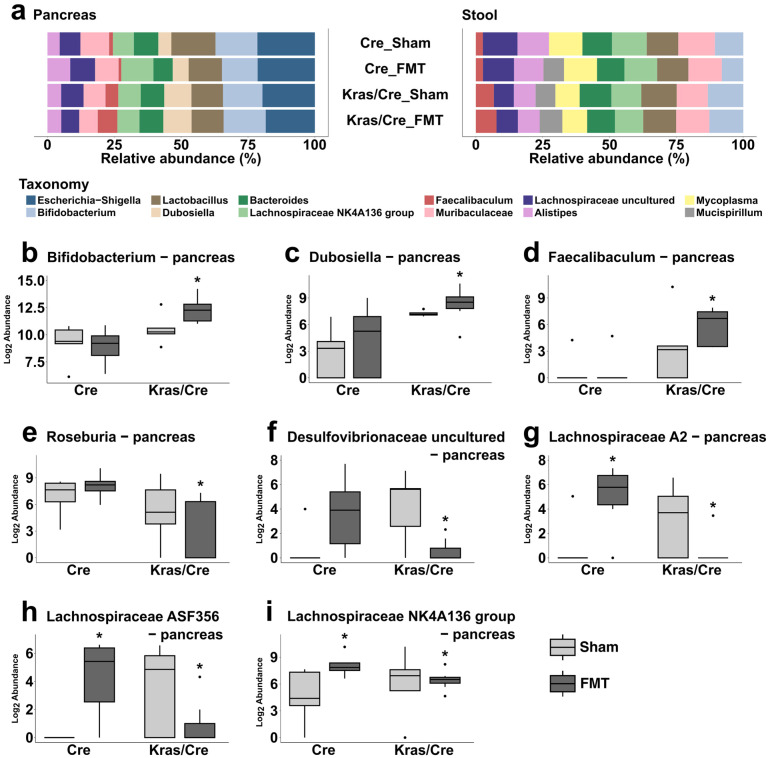
Abundance of bacterial genera after fecal microbiota transplantation associated pancreatic carcinogenesis. Relative abundance of the most common genera in pancreas and stool samples in Kras/Cre mice and Cre mice after FMT and sham treatments (**a**). The differences between tested mice in abundance in pancreatic tissue of *Bifidobacterium* (**b**), *Dubosiella* (**c**), *Faecalibaculum* (**d**), *Roseburia* (**e**), *Desulfovibrionaceae* (**f**), *Lachnospiraceae A2* (**g**), *Lachnospiraceae ASF356* (**h**), and *Lachnospiraceae NK4A136 group* (**i**). The median abundance (%) is indicated by a black line. The box represents the interquartile range. The whiskers extend to the upper adjacent value and the lower adjacent value and dots represent outliers. Statistically significant differences (*p* value < 0.05) between mice are marked * (for details see Table S17). Kras/Cre—mice with Kras mutation, Cre—mice without Kras mutation, FMT—fecal microbiota transplantation.

Next, we assessed the effect of FMT on the bacterial composition and abundance at the genus level in pancreatic and stool samples. The most common genera found in pancreas and stool samples are presented in Figure 5a. In pancreas samples, *Escherichia-Shigella* was the most common genera detected, while in stool samples the most common was *Muribaculaceae* (Figure 5a, Tables S15 and S16).

#### 3.7.1. Fecal Microbiota Transplantation Alters the Pancreatic Bacterial Genera Abundance in Mice Genetically Predisposed to PDAC

We aimed to identify significant differences in the pancreatic abundance of genera between mice with and without Kras mutation as well as differences related to the FMT (Tables S11 and S13, Figure 5b–i). Three observations were noted.

First, the abundance of three genera, *Bifidobacterium*, *Dubosiella*, and *Faecalibaculum*, increased in Kras/Cre mice compared to Cre mice but did not differ between the FMT and sham arms (Figure 5b–d). Secondly, the abundance of two genera, *Roseburia* and *Desulfovibrionaceae uncultured*, decreased after FMT in Kras/Cre mice compared to Cre mice (Figure 5e,f). Finally, compared to sham treatment, after FMT the abundance of *Lachnospiraceae A2, Lachnospiraceae NK4A136 group*, and *Lachnospiraceae ASF356* decreased in Kras/Cre mice, while increasing in Cre mice (Figure 5g–i, Table S18). This suggests that FMT from PDAC mice may alter the pancreatic abundance of several bacterial genera.

#### 3.7.2. Fecal Microbiota Transplantation Alters the Stool Bacterial Genera Abundance in Mice Genetically Predisposed to PDAC

We explored if there were significant alterations in the stool genera abundance related to the FMT in mice with a Kras mutation.

**Figure 6:**
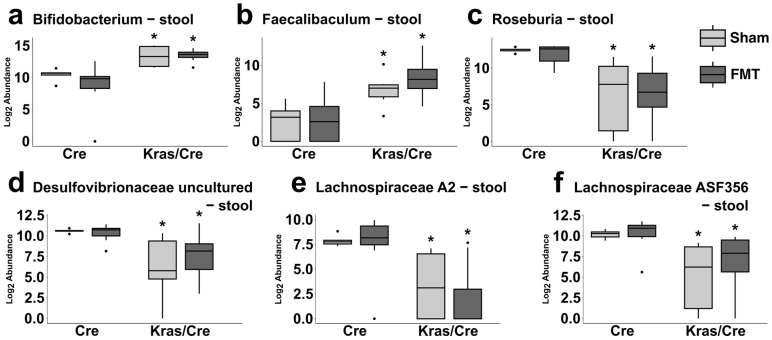
Differences between Kras/Cre mice and Cre mice after fecal transplantation and sham treatments in abundance in stool of bacterial genera. Relative abundance of *Bifidobacterium* (**a**), *Faecalibaculum* (**b**), *Roseburia* (**c**), *Desulfovibrionaceae uncultured* (**d**), *Lachnospiraceae A2* (**e**), *Lachnospiraceae ASF356* (**f**). The median abundance (%) is indicated by a black line. The box represents the interquartile range. The whiskers extend to the upper adjacent value and the lower adjacent value and dots represent outliers. Statistically significant differences (*p* value < 0.05) between mice are marked * (for details see Table S20). Kras/Cre mice—mice with Kras mutation, Cre mice—mice without Kras mutation, FMT—fecal microbiota transplantation.

First, we analyzed the stool abundance of genera that were changed in pancreatic samples. We found that the abundance of *Bifidobacterium* and *Faecalibaculum* increased in Kras/Cre mice compared to Cre mice, but it was not different between FMT and sham arms (Figure 6a,b). Secondly, the abundance of *Roseburia, Desulfovibrionaceae uncultured*, *Lachnospiraceae A2,* and *Lachnospiraceae ASF356* decreased in Kras/Cre mice after both FMT and sham treatments compared to Cre mice (Figure 6c–f). These findings are consistent with the observations made in pancreatic samples.

Furthermore, the assessment of all bacterial genera in stool samples revealed additional alterations in microbiota abundances (Table S19). Specifically, compared to the sham treatment, FMT in both Cre and Kras/Cre mice resulted in the detection of *Alloprevotella*, *Rikenellaceae RC9 gut group*, *Lachnospiraceae unclassified*, *Halomonas*, and *Brachyspira*. Moreover, there were only two genera—*Odoribacter* and *Prevotellaceae UCG-001*, that significantly changed in Kras/Cre mice after FMT compared to sham treatment.

Considering all the data together, it can be suggested that FMT from mice with PDAC may alter the abundance of several genera in both pancreatic and stool samples. Specifically, in both stool and pancreatic samples, we found an increase in the abundance *of Bifidobacterium* and *Faecalibaculum* after FMT in mice with a Kras mutation, which was not observed in Cre mice. More importantly, in mice with a Kras mutation, the observed decrease in pancreatic and stool abundance of *Roseburia*, *Desulfovibrionaceae, Lachnospiraceae A2,* and *Lachnospiraceae ASF356* suggests their possible role in pancreatic carcinogenesis. Indeed, in mice without a Kras mutation, the levels of these genera remained unchanged or increased with FMT.

### 3.8. The Microbiota Alpha and Beta Diversities in Fecal Microbiota Transplantation-Associated Pancreatic Carcinogenesis

**Figure 7:**
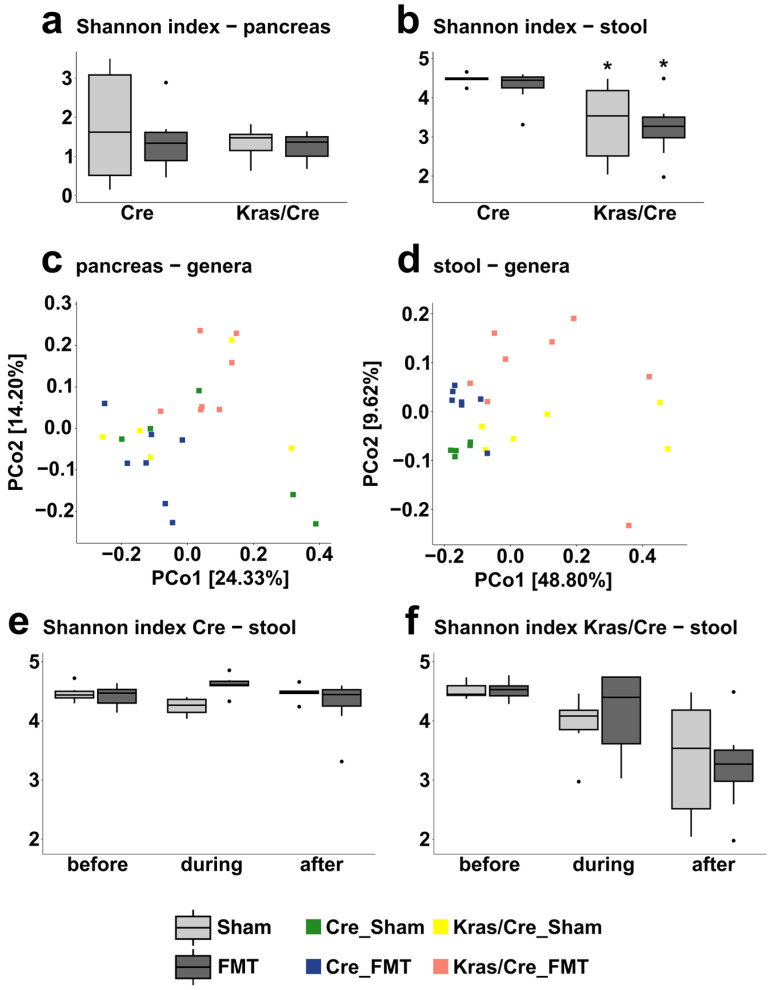
Bacterial microbiota diversity in fecal microbiota transplantation associated pancreatic carcinogenesis. Alpha diversity index (Shannon index) of the microbiota in pancreas (**a**) and stool (**b**) samples in Kras/Cre mice and Cre mice after fecal microbiota transplantation and sham treatments. Principal coordinate analysis (PCoA) plot results based on Bray–Curtis dissimilarity distance at the genera level in Kras/Cre mice and Cre mice after fecal microbiota transplantation and sham treatments in pancreas (**c**) and stool (**d**) samples. Bacterial stool alpha diversity changes (Shannon index) over time after fecal microbiota transplantation in Cre mice (**e**) and Kras/Cre mice (**f**). (**a**,**b**,**e**,**f**): The median is indicated by a black line. The box represents the interquartile range. The whiskers extend to the upper adjacent value and the lower adjacent value and dots represent outliers. Statistically significant differences (*p* value < 0.05) between mice are marked * (for detail see Table S22). Kras/Cre—mice with Kras mutation, Cre—mice without Kras mutation, FMT—fecal microbiota transplantation.

The diversity of the microbial community in pancreas samples, as reflected by the Shannon index, did not significantly change after sham and FMT treatments in Cre mice and Kras/Cre mice (Figure 7a, Table S15). However, differences were found in stool microbiota alpha diversity in Kras/Cre mice after FMT and sham treatments, compared to Cre mice. The diversity index was lower in Kras/Cre mice than in Cre mice. In addition, the diversity index was lower in Kras/Cre mice after FMT, compared to Kras/Cre mice after sham treatment (Figure 7b, Table S15).

For further evaluation of the differences and similarities between pancreatic and stool microbiota after FMT and sham treatments in all study groups, we analyzed the PCoA based on the Bray–Curtis dissimilarity distance. The PCoA plot of pancreatic samples did not reveal any clusters (Figure 7c). In the analysis of fecal samples on a genus level, PCo1 and PCo2 explain 48.80% and 9.62% of the variance, respectively. As presented in Figure 7d, there was no clear separation of the samples, although it can be noted that samples from Cre mice group together, whereas samples from Kras/Cre exhibit greater dispersion. The proximity of samples within the Cre sham and Cre FMT groups suggests their low intra-group variability, while the large spread of Kras/Cre sham and Kras/Cre FMT groups indicates significant intra-group diversity. These findings are in line with the results of the alpha diversity analysis.

As we observed, decreased stool alpha diversity in mice with advanced pancreatic changes after FMT from PDAC, we then investigated whether these changes occur at the late stage of the disease or progressively develop during carcinogenesis. To show the fluctuations of bacterial stool diversity, we collected stools from all experimental mice before, during, and after stimulation with FMT from PDAC. In control Cre mice, small fluctuations without significant differences in bacterial alpha diversity were observed after FMT and sham treatments over time (Figure 7e). In contrast, in Kras/Cre mice, we noticed a gradual decrease in bacterial diversity after both FMT and sham treatments that was accompanied by progress in carcinogenesis from a healthy pancreas to cancer (Figure 7f). This suggests that stimulation with FMT reduces stool bacterial diversity only in the presence of oncogenic Kras mutation and the stool microbiota diversity gradually decreases with the promotion and progression of pancreatic cancer.

## 4. Discussion

In this study, we found that pancreatic carcinogenesis, induced by chronic inflammation in the presence of an oncogenic Kras mutation, is accompanied by alterations in the abundance of pancreatic bacterial microbiota, as well as more pronounced gut dysbiosis. This dysbiosis is characterized by a reduction in alpha bacterial community diversity and a reshaping of bacterial taxonomic composition and abundance. The presence of gut dysbiosis only in mice with a Kras mutation that developed advanced pancreatic lesions and the lack of significant microbiota differences in mice without an oncogenic Kras mutation even after inflammatory stimulation, indicate the crucial role of a genetic predisposition to PDAC. The microbiota changes progress with carcinogenesis more rapidly due to inflammation. Furthermore, we found that tumor-associated stool dysbiosis may influence pancreatic carcinogenesis and cause further microbiota alterations. Fecal microbiota transplanted from mice with PDAC to mice with a Kras mutation but without pancreatic changes accelerated pancreatic tumorigenesis, reduced stool bacterial diversity, and altered the microbiota composition in both stool and pancreatic samples. Overall, the most pronounced change was the reduction in the levels of bacteria known to produce short-chain fatty acids (SCFAs) which have anti-inflammatory properties.

The use of a PDAC animal model gives us the opportunity to test different aspects of disease initiation, progression, and treatment. However, all available models have limitations, and the development and progression of the disease vary across animal models, even when using inbred strains with similar genetic backgrounds. Several factors may contribute to the variability of models and influence disease manifestation [28]. To investigate the influence of external stimuli on the development of PDAC, it is important to select the best animal model of PDAC, as well as the best time to start and stop treatments as the disease progresses with different kinetics in each model [28]. So far, data on intratumoral and stool microbiota diversity and composition in experimental models of PDAC have been scarce. Alterations in microbiota were analyzed in spontaneous Kras G12D expressing mouse model (KC) with pre-invasive PanIN formation, and in Kras and Trp53 expressing mouse models (KPC) with invasive PDAC development, and in an orthotopic PDAC model (using KPC-derived tumor cells) [11,17,18,20,22]. We utilized mice in which a tamoxifen-inducible Cre recombinase is expressed in differentiated acinar cells by a full-length mouse pancreatic elastase I gene promoter. The mice exhibit tamoxifen-independent Cre activity after birth specifically in acinar cells with high levels of elastase expression. In the absence of tamoxifen, Cre activity was restricted to well-differentiated acinar cells [23,24,25]. These mice were crossed with mice that possess the conditional knock-in mutant KrasG12D driven by its promoter, silenced by transcriptional STOP cassette. The STOP sequence prevents the expression of mutant Kras, but it is flanked by LoxP sites so that it can be recognized and excised by Cre recombinase [23,24,25]. The Ras levels differ due to the LoxP-Stop-LoxP sequence independently of tamoxifen administration. Nevertheless, the Ela-Cre mice were used as controls as they did not develop pancreatic tumors [23,24,25]. As it has been previously described, this mouse model with the expression of oncogenic Kras G12D mutation in acinar cells is suitable to test the effects of environmental factors such as inflammation on pancreatic carcinogenesis [23,24,25,28,29]. Based on previous data, the activation of an oncogenic Kras mutation occurs upon overstimulation by inflammation-inducing factors, through an NF-κB-mediated positive feedback mechanism involving Cox-2, which amplifies Ras activity to pathological levels. The expression of active NF-κB kinase 2 or Cox-2, or treatment with LPS, initiated chronic inflammation and PanINs only in mice expressing oncogenic Kras mutation [23,24,25]. This model fully resembles human carcinogenesis, including features of chronic inflammation, the formation of precancerous lesions, and cancer.

In this study, chronic inflammation accelerated the development of PDAC in the presence of the Kras mutation, which was accompanied by changes in stool and pancreatic microbiota. The alterations in microbiota composition were related to the mice’s genotype, older age, and inflammation. Specifically, they were observed only in mice with a Kras mutation that developed PDAC/PanINs with older age or after inflammatory stimulation. Pulshalkar et al. also did not find differences in the pancreatic and stool bacterial profiles between KC mice and wild-type mice at a young age [11]. In addition, Kaune et al. revealed differences in gut microbial community composition related to the genotype, age, and gender in KC and KPC models [22]. Taken together, these findings suggest that changes in the microbiota depend on multiple factors, including genetic profile, the co-existence of inflammation, and the age of the subject.

Most published studies characterize the microbiota in patients with advanced PDAC. Several bacteria were found to be enriched in PDAC tumors such as *Proteobacteria* (*Gammaproteobacteria*), *Firmicutes*, *Fusobacterium*, *Lactobacillus*, and *Bacteroides* (Cruz) [6,11,17,22]. We found that the pancreas harbors its own microbiome that changes with tumor development. The phyla with the highest abundance in pancreatic samples were *Proteobacteria*, *Firmicutes*, and *Actinobacteriota*. The development of PDAC/PanIN was accompanied by an increase in the pancreatic abundance of *Actinobacteriota* and *Bifidobacterium* genera that belong to *Actinobacteriota.* However, no significant differences in pancreatic alpha and beta microbiota diversities were found. Our results are consistent to some extent with the observations of other researchers. In the study of Pfisterer et al., KPC tumors contained significantly more bacterial components and exhibited a different bacterial composition than in healthy pancreatic tissue. Although the alpha diversity metrics did not differ between KPC tumors and healthy pancreas, they found differences in microbial composition based on beta diversity analyses having observed an increase in the *Gammaproteobacteria* abundance [17].

In concordance with previous studies, we found that the fecal microbiome in PDAC is significantly different from non-PDAC microbiota [11,17,22]. A decrease in the stool alpha microbiota diversity index accompanied the development of PDAC, and stool beta diversity differed between mice that developed PDAC and those that did not. In addition, mice with advanced pancreatic presented with increased stool *Actinobacteriota* abundance, but with decreased abundance of other phyla such as *Verrucomicrobiota*, *Desulfobacterota*, *Firmicutes*, as well as genera including *Butyricicoccus*, *Clostridia UCG.014*, and *Roseburia*. In the KC model, the increase in stool *Actinobacteria* and *Bifidobacterium* was observed with the progression of pancreatic dysplasia [11]. In the KPC model, different *Bacteroides* were found to be increased compared to the feces of control mice [17]. In stool samples of PDAC patients, a significant increase in *Proteobacteria*, *Actinobacteria*, and *Fusobacteria* was observed [30]. Furthermore, a reduction in gut bacterial diversity was also found in an animal model of cerulein-induced chronic pancreatitis, characterized by lower levels of *Firmicutes* and higher levels of *Bacteroides*, *Actinobacteria*, and *Verrucomicrobiota* [31].

Moreover, we found that stool dysbiosis may influence the development of PDAC and cause further microbiota alterations. FMT from mice with PDAC into mice with a Kras mutation stimulated pancreatic carcinogenesis and was accompanied by a gradual decrease in stool alpha microbiota diversity with the progression of tumorigenesis. In addition, in mice with a Kras mutation after FMT, but not in mice without a Kras mutation, we found an increase in the pancreatic and stool abundances of *Actinobacteriota*, *Bifidobacterium* and *Faecalibaculum*, as well as a decrease in some bacteria, including *Desulfovibrionaceae uncultured*, *Lachnospiraceae A2* and *Lachnospiraceae ASF356*, and *Roseburia*. The importance of FMT in PDAC pathogenesis has also been highlighted by others [11,21,32]. In a study by Genton et al., changes in microbiota composition were observed in patients with PDAC and mice after FMT from these patients, characterized by a lower proportion of *Lachnospiraceae species*, *Alistipes obesi, Coriobacteriaceae*, and a higher proportion of *Clostridium scindens*, *Clostridium bolteae,* and *Phascolarctobacterium faecium* [25]. In a KC model, *Bifidobacterium pseudolongum* accelerated oncogenesis in a TLR-dependent manner [11]. Riquelme et al. found that tumor growth was decreased in mice with FMT from long-term survival PDAC patients compared to mice with FMT from short-term survival patients [21].

Although we did not analyze immune cell infiltrations, there is evidence that microbiota influences pancreatic carcinogenesis through altering the immune response. It can regulate dendritic cells, monocytes/macrophages, natural killer cells, CD8+ T cells, and CD4+ T cells to stimulate immune response [33]. CD8+ T cells play an important role in anticancer immunity. Mice with PDAC-enriched CD8+ T cells survive longer. Similarly, in patients with PDAC, tissue infiltration with CD8+ T cells is associated with longer survival [34,35]. Moreover, the CD8+ cell infiltration can be increased by specific bacteria. In melanoma, the high abundance of *Ruminoccocus*, *Faecalibacterium,* and *Clostridium* was related to improved function of CD4+ and CD8+ T cells as well as better antitumor effects of immune checkpoint inhibitors [36]. In our study, the stool abundance of *Clostridia UCG-014* did not change significantly in mice that did not develop advanced pancreatic lesions and decreased with the promotion of carcinogenesis. In a study by Riquelme et al., long-term PDAC survivors had higher intratumoral bacterial diversity, a different microbiome signature, and an increased number of CD8^+^ T-cells compared to short-term survivors [21]. Moreover, through experiments with human-to-mice FMT, they observed effects on the tumor microbiome, tumor growth, and tumor immune infiltration. In comparison to the mice with FMT from short-term survival PDAC patients, the mice that received FMT from long-term survival donors exhibited a significant reduction of tumor growth accompanied by a significantly higher number of CD8+ cells and activated T cells and higher serum levels of INFγ and IL-2. In addition, the depletion of CD8+ blocked the anti-tumoral effect induced by FMT from long-term survival donors [21].

In addition, initial animal experiments have shown that antibiotic-mediated bacterial depletion delayed tumor growth [10,11,22]. In mice with concomitant Kras mutation and partial loss of PTEN tumor suppression, bacterial ablation with antibiotics resulted in a decreased rate of PDAC development. In wild-type mice, oral antibiotic treatment resulted in a 50% reduction in orthotopic pancreatic tumor size [21,27]. We used antibiotics before FMT based on the previous data [21,26,27]. Given the relatively long duration of the experiment, a two-week course of antibiotics should have no effect on cancer progression and microbiota alteration associated with the tumor. Indeed, both mice with Kras mutation receiving FMT and sham treatments were administrated with antibiotics, but the incidence of pancreatic cancer was higher in the FMT group. Moreover, no advanced pancreatic changes as well as significant microbiota changes were observed in mice without Kras mutation after both FMT and sham treatments.

It can be suggested that Kras overstimulation is a major driving force behind tumor growth and may be supported by dysbiosis and bacteria-delivered metabolites, and other pro-inflammatory and pro-oncogenic factors. It is noteworthy, that in our study, several genera such as *Alloprevotella, Oscillospiraceae, Butyricicoccus*, *Roseburia*, and *Lachnospiraceae* were found to be decreased in stool or not detected in the pancreas after the initiation of pancreatic carcinogenesis. These genera are involved in the production of SCFAs such as butyrate through the fermentation of dietary fiber and other substrates [37,38]. The butyrate is mainly produced by *Firmicutes*, which was the most abundant phyla in stool samples and was reduced significantly in mice with PanIN/PDAC. In patients with PDAC, compared to healthy controls, a reduction in SCFA-producing bacteria with anti-inflammatory properties and an increase in LPS-producing bacteria were observed [10]. Similar observations were noted in chronic pancreatitis [39]. Although we did not analyze SCFAs in this study, it has been suggested that SCFAs may be involved in cancer development through the modulation of inflammation, cell proliferation, and immune response [37,38,40,41,42]. SCFAs may control the production of cathelicidin-related antimicrobial peptides by pancreatic cells which results in an intrapancreatic macrophage switch from an inflammatory to a regulatory phenotype as well as an induction of dendritic cells and regulatory T cells within the pancreas [41]. The decrease of SCFAs is involved in the activation of the NF-kB pathway and GTPases, including Ras [38]. The role of SCFAs can be particularly important in this mouse model, in which NF-κB-mediated positive feedback mechanism leads to the overstimulation of oncogenic Kras. Taken together, the decrease in the levels of SCFAs-producing bacteria observed in our study and the results of previous research on the potential mechanism of SCFAs in cancer suggest their crucial role in the promotion and progression of pancreatic carcinogenesis.

In addition, the observation of a decreased abundance of the *Firmicutes* phylum and other above-mentioned genera which are sources of SCFAs, gives insight into data suggesting their influence on the effectiveness of chemotherapy [40]. Butyrate enhances gemcitabine effectiveness against human pancreatic cancer cell lines, mainly by inducing apoptosis and modulating the tumor microenvironment in the orthotopic mouse model of PDAC [42]. Recently, a clinical trial found that *Lachnospiraceae*-enriched FMT enhanced anti-programmed cell death protein 1 immunotherapy and augmented CD8^+^ T cell infiltration in melanoma patients [43]. It is noteworthy that SCFAs enhance the anti-tumor activity of cytotoxic T lymphocytes (CTLs) and chimeric antigen receptor (CAR) T cells through metabolic and epigenetic reprogramming [44].

Another observation from our study was a decrease in the stool abundance of *Lachnoclostridium* in mice with PDAC. In patients with unresectable hepatocellular carcinoma who responded to immunotherapy, an enrichment of *Lachnoclostridium* was found in fecal samples and was associated with better overall survival [45]. Furthermore, the increase in fecal *Lachnoclostridium* was associated with the presence of intratumoral tertiary lymphoid structures, which are linked to a favorable response to immunotherapy [46]. Bacterial genera such as *Lachnoclostridium* that we found to be significantly altered with pancreatic tumorigenesis may be useful in diagnosis and may serve as predictive biomarkers of response to therapy.

The exact mechanisms by which microbiota can reach pancreatic tissue are still unknown. It has been proposed that microbiota can migrate from the gastrointestinal tract through the major ampulla of the duodenum or via the mesenteric venous and lymphatic system of the gut, particularly when affected by defective intestinal permeability [7,11,20,47]. Kohi et al. found that patients with PDAC had increased levels of *Bifidobacterium* in duodenal fluid compared with control subjects and patients with pancreatic cysts [48]. We found an increase in *Bifidobacterium* in the stool samples of mice with PDAC and pancreas samples after FMT. Additionally, a similar pattern of bacteria alterations in both pancreas and stool samples as well as the genera changes related to the administration of FMT suggest the existence of the intestinal-pancreatic axis. Bacteria-derived SCFAs that regulate immune responses in the pancreas may play crucial roles in this axis [49].

The pancreatic exocrine function has been shown to contribute significantly to the composition of intestinal microorganisms in individuals without pancreatic disease [50]. Pancreatic exocrine insufficiency is a common condition in patients with PDAC [51]. In addition, in fecal samples from patients undergoing pancreaticoduodenectomy, *Klebsiella* spp. has been shown to be enriched, while *Faecalibacterium prausnitzii* and *Roseburia* spp. have been shown to be decreased. Both of these species are thought to have potential anti-inflammatory effects [52]. On the other hand, pancreatic exocrine insufficiency is known to induce bacterial dysbiosis in experimental studies [53,54]. Intestinal dysbiosis can be present in patients with compromised pancreatic exocrine function [55]. In addition, Ahuja et al. found that the deletion of the acinar Ca^2+^ channel Orai1 in mice, a channel involved in pore formation in the cellular membrane during acinar cell exocytosis for secretion of pancreatic juice, resulted in high mortality secondary to bacterial overgrowth. The bacterial overgrowth shifted the microbiota to a pro-inflammatory phenotype due to decreased secretion of antimicrobial peptides [56]. In contrast, Pfisterer et al. concluded that the early shift in the fecal microbiome in KPC and KC mice is most likely driven by genotype and cancer progression, rather than by pancreatic exocrine dysfunction [17].

The “driver-passenger” paradigm has been suggested in colorectal cancer, which proposes that the colonization and active invasion of the “driver(s)” bacteria cause damage, allowing other commensals, known as “passengers”, or their by-products, i.e., metabolites, to pass through the epithelium. The bacteria and their products may induce epithelial DNA damage and epigenetic alterations, leading to tumorigenesis. The tumor generates a microenvironment that, in turn, promotes the proliferation of “passenger bacteria” that are better adapted to the conditions in and around cancer cells [57]. The tumor microenvironment may impact microbiota composition through the induction of bacterial proliferation and modulation of compensatory and adaptive mechanisms within the pancreas and intestine, which further influence cancer progression.

## 5. Conclusions

Pancreatic carcinogenesis induced by chronic inflammation in the presence of an oncogenic Kras mutation is associated with a reduction in stool bacterial diversity and significant changes in the microbiota composition in both stool and pancreas samples. Moreover, tumor-associated stool dysbiosis may influence the initiation and progression of PDAC and cause further microbiota alterations. FMT from mice with a Kras mutation and PDAC to mice with a Kras mutation without pancreatic changes accelerated pancreatic tumorigenesis, reduced stool bacterial diversity, and altered the microbiota composition in both stool and pancreas samples. The most pronounced effect was the reduction in the levels of bacteria known to produce short-fatty acids with anti-inflammatory properties. A genetically engineered inducible mouse model with a pancreas-specific expression of a Kras mutation is a suitable model for microbiome studies. Our data contribute to a better understanding of the role of chronic inflammation, bacterial dysbiosis, and genetic predisposition in PDAC development. The findings provide a basis for future research on the mechanisms of specific interactions between the microbiome and PDAC and should be beneficial in future studies on more personalized and effective cancer care strategies.

## Data Availability

The original contributions presented in this study are included in the article/Supplementary Materials. Further inquiries can be directed to the corresponding author.

## Supplementary Materials

The following supporting information can be downloaded at: https://www.mdpi.com/article/10.3390/cells14050361/s1, Supplementary Materials S1: Methods. Library preparation, sequencing, and bioinformatics analysis. Supplementary Materials S2: Results. Figure S1. Representative histopathology images of the pancreas in inflammation-induced pancreatic carcinogenesis. Figure S2. Representative histopathology images of the pancreas in fecal microbiota transplantation experiment. Table S1. Histopathology results. Table S2. The prevalence and abundance of phyla in pancreas samples of mice with Kras mutation and mice without Kras mutation in inflammation-associated pancreatic carcinogenesis. Table S3. The prevalence and abundance of phyla in stool samples of Kras mutation and mice without Kras mutation in inflammation-associated pancreatic carcinogenesis. Table S4. The significant differences (*p* value) for comparison in the phyla abundances are presented in Figure 1. Table S5. The prevalence and abundance of the most common genera in pancreas samples of mice with Kras mutation and mice without Kras mutation. Table S6. The prevalence and abundance of the most common genera in stool samples of mice with Kras mutation and mice without Kras mutation. Table S7. The significant differences (*p* value) for comparison in the genera abundances are presented in Figure 2. Table S8. The percentage of pancreatic samples with genera not detected in inflammation-associated pancreatic carcinogenesis. Table S9. The alpha diversity indexes (Shannon index and Simpson index) of pancreatic and stool microbiota mice with Kras mutation and mice without Kras mutation in inflammation-induced carcinogenesis. Table S10. The comparison of microbiota alpha diversity indexes (Shannon index, Simpson index) in inflammation-induced carcinogenesis (*p* values) A. pancreas samples, B. stool samples. Table S11. The significant differences (*p* value) for comparison in the alpha diversity index are in Figure 3. Table S12. The prevalence and abundance of phyla in pancreas samples between Cre mice and Kras/Cre mice after fecal microbiota transplantation and sham treatment. Table S13. The prevalence and abundance of phyla in stool samples between Cre mice and Kras/Cre mice after fecal microbiota transplantation and sham treatment. Table S14. The significant differences (*p* value) for comparison in the phyla abundances are presented in Figure 4. Table S15. The prevalence and abundance of the most common bacterial genera in pancreas samples in the fecal microbiota transplantation associated pancreatic carcinogenesis. Table S16. The prevalence and abundance of the most common bacterial genera in stool samples in the fecal microbiota transplantation associated pancreatic carcinogenesis. Table S17. The significant differences (*p* value) for comparison in the genera abundances are presented in Figure 5. Table S18. The differences in genera abundance in pancreas samples between Cre mice and Kras/Cre mice after fecal microbiota transplantation and sham treatment. Table S19. The differences in genera abundance in stool samples between Cre mice and Kras/Cre mice after fecal microbiota transplantation and sham treatment. Table S20. The significant differences (*p* value) for comparison in the genera abundances are presented in Figure 6. Table S21. The diversity index (Shannon index) in Kras/Cre mice and Cre mice after fecal microbiota transplantation. Table S22. The significant differences (*p* value) for comparison in the alpha diversity index are in Figure 7.

## Abbreviations

The following abbreviations are used in this manuscript:

PDAC	Pancreatic ductal adenocarcinoma
FMT	Fecal microbiota transplantation
PanIN	Pancreatic intraepithelial neoplasia
Kras/Cre	Transgenic mice with Kras G12D mutation
Cre	Transgenic mice without Kras G12D mutation
CER	Cerulein
SAL	Saline
