# Supplementary material for: Inflammatory Stimuli and Fecal Microbiota Transplantation Accelerate Pancreatic Carcinogenesis in Transgenic Mice, Accompanied by Changes in the Microbiota Composition
Source: Cells. 2025 Feb 28;14(5):361. doi: 10.3390/cells14050361 (PMC11898920; doi:10.3390/cells14050361)
Supplement: Supplementary file 1 [file cells-14-00361-s001.zip › Supplementary Material revised.pdf]

Supplementary Material S1.

Methods. Library preparation, sequencing and bioinformatics analysis.

Metagenomic analysis of bacterial and archaeal populations was performed based on the hypervariable V3-V4 region of the 16S rRNA gene. Specific primer sequences 341F and 785R were used to amplify the selected region and prepare the library (16S analysis). PCR was performed using Q5 Hot Start High-Fidelity 2X Master Mix, reaction conditions according to the manufacturer's recommendations. Sequencing was performed on a MiSeq device, using paired-end (PE) technology, 2x300nt, using the v3 Illumina kit. Detailed information is available on the websites of reagent manufacturers.

Automatic preliminary data analysis was performed on a MiSeq sequencer using MiSeq Reporter (MSR) software. The analysis consisted of two stages: 1. automatic demultiplexing of samples, 2. generation of fastq files containing raw reads.

Bioinformatics analysis ensuring classification of reads to the species level was performed using the QIIME 2 software package based on the 138 Silva reference sequence database. The DADA2 package was also used, which allowed for the identification of sequences of biological origin from those newly created in the sequencing process. This package was also used to extract unique sequences of biological origin, i.e. the so-called ASV sequence (amplicon sequence variant). The analysis consisted of the following stages:
1. Quality control of readings:
- analysis of the error profile of individual samples and dynamic generation of parameters for quality control (FIGARO tool) ´
- carrying out quality control based on the maximum expected errors of the sample
2. data pre-processing using the Cutadapt tool: ˙
- removal of adapter sequences
- rejection of too short reads (minimum length 30 nt)
3. selection of unique ASV sequences (using the DADA2 package) by:
- filtering out sequences containing errors arising during the sequencing process (denoising)
- combining paired reads - in order to increase the accuracy of sequencing, they are performed in paired-end mode, which means that at later stages of analysis, corresponding forward and reverse reads must be combined
- dereplications - merging identical, unique sequences while maintaining the number of their occurrences and quality profile

- chimera filtering - getting rid of constructs resulting from incorrect sequence assembly during PCR

4. taxonomy assignment to ASV sequences based on the Silva reference database, using a hybrid approach:

- comparison of ASV sequences against the database in search of identical reference sequences (vsearch)
- unusual sequences remaining after the previous step are classified based on methods using machine learning (sklearn)

5. creating a phylogenetic tree

- performing alignment using the MAFFT algorithm
- construction of a phylogenetic tree using the FastTree method

Additional extended bioinformatics analysis was performed using the R program and the phyloseq and vegan packages.

Supplementary Material S2. RESULTS.

Table S1. Histopathology results.

Mice	Treatment	Time (days)	PanIN3/PDAC absent (%)	PanIN3/PDAC present (%)
Inflammation-induced pancreatic carcinogenesis				
Cre	Saline	30	100	0
Cre	Saline	120	100	0
Cre	Cerulein	30	100	0
Cre	Cerulein	120	100	0
Kras/Cre	Saline	30	91	9
Kras/Cre	Saline	120	31,5	68,5
Kras/Cre	Cerulein	30	37,5	62,5
Kras/Cre	Cerulein	120	5	95
Fecal microbiota transplantation and sham treatment				
Cre	Sham		100	0
Cre	Fecal microbiota transplantation		100	0
Kras/Cre	Sham		20	80
Kras/Cre	Fecal microbiota transplantation		0	100

PanIN – pancreatic intraepithelial neoplasia, PDAC – pancreatic ductal adenocarcinoma, Kras/Cre – mice with Kras mutation, Cre – mice without Kras mutation

Figure S1. Representative histopathology images of the pancreas in inflammation-induced pancreatic carcinogenesis.

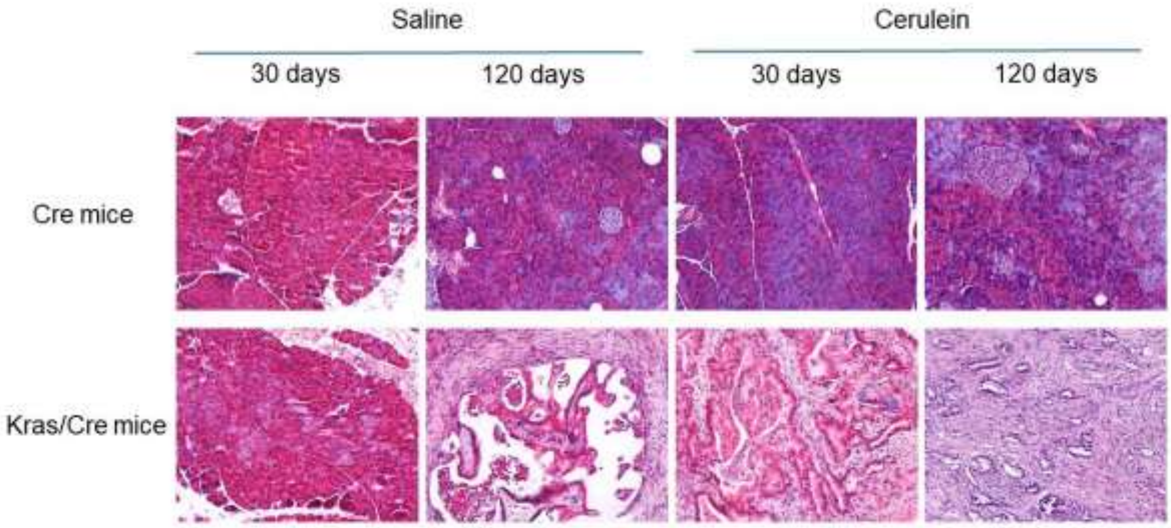

Kras/Cre – mice with Kras mutation, Cre – mice without Kras mutation.

The histopathology images (H&E, 200x) of pancreas of mice without Kras mutation that show no advanced pancreatic changes after saline and cerulein injections. In contrast, the mice with Kras mutation developed advanced pancreatic changes including pancreatic intraepithelial neoplasia (PanIN) and pancreatic ductal adenocarcinoma (PDAC),120 days after saline injections, and 30 and 120 days after cerulein injections, but not 30 days after saline injections.

Figure S2. Representative histopathology images of the pancreas in fecal microbiota transplantation experiment.

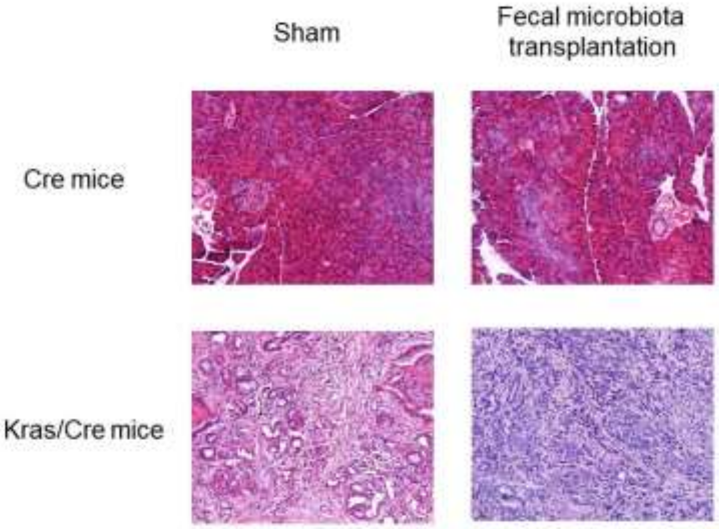

Kras/Cre – mice with Kras mutation, Cre – mice without Kras mutation.

The pancreas histopathology (H&E, 200x) of mice without Kras mutation (Cre mice) shows no advanced pancreatic changes after sham treatment and fecal microbiota transplantation (FMT). In contrast, the mice with Kras mutation developed advanced pancreatic changes (pancreatic intraepithelial neoplasia, PanIN and pancreatic ductal adenocarcinoma, PDAC), after sham and FMT treatments.

Table S2. The prevalence and abundance of phyla in pancreas samples of mice with Kras mutation and mice without Kras mutation in inflammation-associated pancreatic carcinogenesis

Cre – mice without Kras mutation, Kras/Cre – mice with Kras mutation, Sal – saline, CER - cerulein

2	**Relative abundance - pancreas phyla**											
3	% reads											
4	Group	Actinobact	Bacteroidc	Campiloba	Cyanobact	Deferribac	Desulfoba	Firmicutes	Patescibac	Proteobac	Spirochaet	Verrucomicrobiota
5	Cre Sal 30 days	11,54063	10,46213	9,585838	4,537797	4,531877	7,639317	15,24891	5,51408	18,55417	6,194581	6,190673
6	Kras/Cre Sal 30 days	13,87321	13,00444	12,07193	2,75953	2,910877	3,156781	15,84358	5,610582	27,18315	3,585916	0
7	Cre Sal 120 days	16,10064	12,16414	9,782311	2,811963	1,642697	5,156888	18,62504	2,82915	30,88717	0	0
8	Kras/Cre Sal 120 days	19,27934	13,96886	10,2913	4,741076	6,623294	1,486306	17,15462	3,601534	20,80704	0	2,04663
9	Cre CER 30 days	14,67751	10,99923	10,39239	6,23864	0	5,864925	18,16324	2,45431	23,74681	2,325387	5,137549
10	Kras/Cre CER 30 days	18,79241	13,75688	11,92508	3,22853	2,301934	0	20,12175	0,79633	22,08378	2,199288	4,79402
11	Cre CER 120 days	13,73779	12,02952	7,680511	5,017816	6,272666	7,346553	16,4035	6,417825	22,64806	0	2,44576
12	Kras/Cre CER 120 days	17,32078	13,36569	16,65436	2,316696	3,617507	1,73706	16,31857	2,423989	24,4321	0	1,813246
13												
14	**Prevalance - pancreas phyla**											
15	%											
16	Group	Actinobact	Bacteroidc	Campiloba	Cyanobact	Deferribac	Desulfoba	Firmicutes	Patescibac	Proteobac	Spirochaet	Verrucomicrobiota
17	Cre Sal 30 days	100	75	75	50	50	75	100	75	100	75	75
18	Kras/Cre Sal 30 days	100	100	80	40	20	20	100	60	100	40	0
19	Cre Sal 120 days	100	75	75	25	25	50	100	25	100	0	0
20	Kras/Cre Sal 120 days	100	100	80	60	60	20	100	40	100	0	20
21	Cre CER 30 days	100	75	75	75	0	50	100	25	100	25	75
22	Kras/Cre CER 30 days	100	100	75	25	25	0	100	25	100	25	25
23	Cre CER 120 days	100	83,33333	66,66667	50	66,66667	66,66667	100	66,66667	100	0	16,66667
24	Kras/Cre CER 120 days	100	100	100	25	50	25	100	25	100	0	25

Table S3. The prevalence and abundance of phyla in stool samples of Kras mutation and mice without Kras mutation in inflammation-associated pancreatic carcinogenesis

Cre – mice without Kras mutation, Kras/Cre – mice with Kras mutation, Sal – saline, CER - cerulein

Relative abundance - stool phyla											
% reads											
Group	Actinobacteriota	Bacteroidota	Campilobacterota	Cyanobacteria	Deferribacterota	Desulfobacterota	Firmicutes	Patescibacteria	Proteobacteria	Spirochaetota	Verrucomicrobiota
Cre Sal 30 days	7,001064265	14,35351239	12,76150485	0,396349859	9,003141964	10,61991241	15,43300811	8,357104428	7,503159931	8,393035718	6,178206072
Kras/Cre Sal 30 days	8,577227594	15,48978	10,84080569	0	7,297974697	10,97074262	16,08090923	7,192294479	9,714118602	6,801203066	7,034944018
Cre Sal 120 days	10,17473256	15,33926316	10,5944073	2,7861481	6,274204683	10,92590918	16,7651996	6,140049331	9,338877516	5,307325019	6,353883534
Kras/Cre Sal 120 days	15,18981284	15,24781867	11,27789464	2,260239962	9,320955542	8,45715026	14,86051907	5,298494834	8,010225698	5,870630981	4,206257505
Cre CER 30 days	8,399735955	14,96550081	11,85767456	0	7,490101234	10,87307093	15,51616889	8,661205486	8,170682881	7,770780903	6,295078375
Kras/Cre CER 30 days	15,34739492	15,78690956	12,47491389	2,125523365	9,111474555	8,213103023	15,75194064	3,298158726	8,579838845	5,157993075	4,1527494
Cre CER 120 days	8,93569457	13,52400432	12,12842364	0,880319645	8,585127197	10,61584037	15,08117026	7,383154338	8,509665048	7,344909811	7,011690807
Kras/Cre CER 120 days	12,38017401	14,98386448	11,58294091	0,326342498	9,822564726	9,842040825	14,98156305	5,964957072	8,928803956	6,688622647	4,498125825

Prevalance - stool phyla											
% reads											
Group	Actinobacteriota	Bacteroidota	Campilobacterota	Cyanobacteria	Deferribacterota	Desulfobacterota	Firmicutes	Patescibacteria	Proteobacteria	Spirochaetota	Verrucomicrobiota
Cre Sal 30 days	87,5	100	100	12,5	100	100	100	100	100	100	100
Kras/Cre Sal 30 days	100	100	100	0	80	100	100	80	100	80	100
Cre Sal 120 days	100	100	100	58,33333333	91,66666667	100	100	91,66666667	100	91,66666667	100
Kras/Cre Sal 120 days	100	100	100	77,77777778	100	100	100	66,66666667	100	100	88,88888889
Cre CER 30 days	100	100	100	0	100	100	100	100	100	100	100
Kras/Cre CER 30 days	100	100	100	62,5	100	100	100	62,5	100	87,5	75
Cre CER 120 days	100	100	100	37,5	100	100	100	100	100	100	100
Kras/Cre CER 120 days	100	100	100	12,5	100	100	100	75	100	100	87,5

Table S4. The significant differences (p value) for comparison in the phyla abundances presented in Figure 1.

Figure 1	P value
b	Kras/Cre CER 30 days vs Kras/Cre Sal 30 days (p=0.030), Kras/Cre Sal 120 days vs Cre Sal 120 days (p=0.0380), Kras/Cre Sal 30 days vs Kras/Cre Sal 120 days (p=0.0108).
c	Kras/Cre CER 30 days vs Cre CER 30 days (p=0.0023), Kras/Cre CER 30 days vs Cre CER 120 days (p=0.0100), Kras/Cre CER 30 days vs Cre Sal 30 days (p=0.0002), Kras/Cre CER 120 days vs Cre Sal 30 days (p=0.0084), Kras/Cre CER 30 days vs Cre Sal 120 days (p=0.0078), Kras/Cre CER 30 days vs Kras/Cre Sal 30 days (p=0.0083), Kras/Cre Sal 120 days vs Cre CER 30 days (p=0.0018), Kras/Cre Sal 120 days vs Cre CER 120 days (p=0.0088), Kras/Cre Sal 120 days vs Cre Sal 30 days (p=0.0002), Kras/Cre Sal 120 days vs Cre Sal 120 days (p=0.0059), Kras/Cre Sal 120 days vs Kras/Cre Sal 30 days (p=0.0070).
d	Kras/Cre CER 30 days vs Cre CER 30 days (p=0.0472), Kras/Cre CER 30 days vs Cre CER 120 days (p=0.0031), Kras/Cre CER 120 days vs Cre CER 120 days (p=0.0048), Kras/Cre CER 30 days vs Cre Sal 30 days (p=0.0476), Kras/Cre CER 30 days vs Cre Sal 120 days (p=0.0387), Kras/Cre CER 30 days vs Kras/Cre Sal 30 days (p=0.0392), Kras/Cre CER 120 days vs Kras/Cre Sal 30 days (p=0.0479), Kras/Cre Sal 120 days vs Cre CER 30 days (p=0.0237), Kras/Cre Sal 120 days vs Cre CER 120 days (p=0.0087), Kras/Cre Sal 120 days vs Cre Sal 30 days (p=0.0256), Kras/Cre Sal 120 days vs Cre Sal 120 days (p=0.0211), Kras/Cre Sal 120 days vs Kras/Cre Sal 30 days (p=0.0212).
e	Kras/Cre CER 30 days vs Cre CER 30 days (p=0.0030), Kras/Cre CER 30 days vs Cre CER 120 days (p=0.0034), Kras/Cre CER 30 days vs Cre Sal 30 days (p=0.0101), Kras/Cre CER 30 days vs Cre Sal 120 days (p=0.0101), Kras/Cre Sal 120 days vs Cre CER 30 days (p=0.0030), Kras/Cre Sal 120 days vs Cre CER 120 days (p=0.0049), Kras/Cre Sal 120 days vs Cre Sal 30 days (p=0.0094), Kras/Cre Sal 120 days vs Cre Sal 120 days (p=0.0093)
f	Kras/Cre CER 30 days vs Cre CER 30 days (p=0.0200), Kras/Cre CER 30 days vs Cre CER 120 days (p=0.0029), Kras/Cre CER 120 days vs Cre CER 30 days (p=0.0454), Kras/Cre CER 120 days vs Cre CER 120 days (p=0.0084), Kras/Cre CER 30 days vs Cre Sal 30 days (p=0.0293), Kras/Cre CER 30 days vs Cre Sal 120 days (p=0.0068), Kras/Cre CER 120 days vs Cre Sal 120 days (p=0.0211), Kras/Cre CER 30 days vs Kras/Cre Sal 30 days (p=0.0281), Kras/Cre Sal 120 days vs Cre CER 30 days (p=0.0046), Kras/Cre Sal 120 days vs Cre CER 120 days (p=0.0004), Kras/Cre Sal 120 days vs Cre Sal 30 days (p=0.0072), Kras/Cre Sal 120 days vs Cre Sal 120 days (p=0.0012), Kras/Cre Sal 120 days vs Kras/Cre Sal 30 days (p=0.0637), Kras/Cre Sal 120 days vs Kras/Cre Sal 30 days (p=0.0212).

Kras/Cre – mice with Kras mutation, Cre mice without Kras mutation, Sal- saline, CER - cerulein

Table S5. The prevalence and abundance of the most common genera in pancreas samples of mice with Kras mutation and mice without Kras mutation.

Cre – mice without Kras mutation, Kras/Cre – mice with Kras mutation, Sal – saline, CER – cerulein

Relative abundance: pancreas genera										
% reads										
Group	Bacteroid	Bifidobact	Coriobact	Dubosiella	Enhydrob	Escherichi	Lachnospi	Lactobacil	Muribacu	Roseburia
Sal_Cre_30 days	6,57004	8,25189	4,48461	9,8532	6,52791	19,7298	9,34469	14,8907	10,4287	9,91839
Sal_Kras/Cre_30 days	12,2494	8,7134	0	6,20447	6,20217	37,2551	4,48291	14,6416	6,49856	3,75244
Sal_Cre_120 days	6,24264	10,531	0	4,0957	13,1967	28,4352	7,67938	12,0387	8,57741	9,20336
Sal_Kras/Cre_120 days	7,28267	18,5794	7,14564	12,4007	7,9676	19,5017	3,4994	12,8549	9,47577	1,2922
CER_Cre_30 days	8,72765	13,7017	2,2443	6,58564	6,16135	23,6919	6,88889	16,4899	7,26242	8,24625
CER_Kras/Cre_30 days	4,69303	15,4943	9,01649	11,6879	6,52844	18,336	5,06872	16,7808	8,35109	4,0432
CER_Cre_120 days	4,87048	11,1203	3,12224	8,68725	8,31814	22,0013	10,9611	11,771	10,3859	8,76227
CER_Kras/Cre_120 days	9,18931	13,2612	6,03905	11,0239	5,12503	22,3642	4,57935	13,7269	10,2109	4,48011

Prevalance: pancreas genera										
%										
Group	Bacteroid	Bifidobact	Coriobact	Dubosiella	Enhydrob	Escherichi	Lachnospi	Lactobacil	Muribacu	Roseburia
Sal_Cre_30 days	75	75	50	75	75	100	75	100	75	75
Sal_Kras/Cre_30 days	66,6667	50	0	33,3333	33,3333	100	16,6667	66,6667	33,3333	16,6667
Sal_Cre_120 days	50	75	0	50	100	100	75	75	75	75
Sal_Kras/Cre_120 days	60	100	60	80	80	100	40	80	80	20
CER_Cre_30 days	75	100	25	50	50	100	50	100	50	75
CER_Kras/Cre_30 days	50	100	75	100	50	100	50	100	75	50
CER_Cre_120 days	50	100	33,3333	83,3333	66,6667	100	83,3333	100	83,3333	66,6667
CER_Kras/Cre_120 days	100	100	50	100	50	100	50	100	100	50

Table S6. The prevalence and abundance of the most common genera in stool samples of mice with Kras mutation and mice without Kras mutation.

Cre – mice without Kras mutation, Kras/Cre – mice with Kras mutation, Sal – saline, CER - cerulein

Relative abundance: stool genera										
% reads										
Group	Alistipes	Bacteroid	Bifidobact	Lachnospi	Lachnospi	Lactobacil	Mucispiril	Muribacul	Odoribact	Prevotellaceae_UCG-0
Sal_Cre_30 days	10,07418	8,839461	2,457235	12,18693	12,05347	12,13726	8,543739	12,30726	9,707871	11,6926
Sal_Kras/Cre_30 days	11,33298	10,79278	4,606793	12,00821	11,85047	11,15925	7,054164	12,51917	8,545665	10,13052
Sal_Cre_120 days	9,007551	10,63218	8,359142	12,55741	11,33569	11,57756	6,218883	12,60732	8,169517	9,534752
Sal_Kras/Cre_120 days	8,250921	10,74942	13,23716	9,629527	8,184069	11,23231	8,126155	12,39724	8,148503	10,0447
CER_Cre_30 days	10,13259	10,34945	4,553537	11,55921	11,57315	12,11826	6,904737	12,75939	8,33527	11,7144
CER_Kras/Cre_30 days	8,412762	11,71977	13,79129	10,43662	7,333791	12,77955	8,337474	13,68587	4,205908	9,296971
CER_Cre_120 days	9,957868	9,26698	4,915576	12,3027	11,82535	11,91076	8,398982	12,05017	8,855889	10,51573
CER_Kras/Cre_120 days	9,625659	10,18141	9,798742	10,90017	9,017953	10,86982	8,632256	12,04196	8,639313	10,29271

Prevalance: stool genera										
%										
Group	Alistipes	Bacteroid	Bifidobact	Lachnospi	Lachnospi	Lactobacil	Mucispiril	Muribacul	Odoribact	Prevotellaceae_UCG-0
Sal_Cre_30 days	100	100	37,5	100	100	100	100	100	100	100
Sal_Kras/Cre_30 days	100	100	60	100	100	80	80	100	100	80
Sal_Cre_120 days	100	100	83,33333	100	100	91,66667	91,66667	100	100	91,66667
Sal_Kras/Cre_120 days	100	100	100	100	100	100	100	100	100	100
CER_Cre_30 days	100	100	75	100	100	100	100	100	100	100
CER_Kras/Cre_30 days	87,5	100	100	100	87,5	100	100	100	50	87,5
CER_Cre_120 days	100	100	75	100	100	100	100	100	100	100
CER_Kras/Cre_120 days	100	100	100	100	100	100	100	100	100	100

Table S7. The significant differences (p value) for comparison in the genera abundances presented in Figure 2.

Figure 2	P value
b	Kras/Cre CER 30 days vs. Kras/Cre Sal 30 days (p=0.0407), Cre Sal 120 days vs. Kras/Cre Sal 120 days (p=0.0436), Kras/Cre Sal 30 days vs. Kras/Cre Sal 120 days (p=0.0044).
c	Cre CER 30 days vs. Kras/Cre CER 30 days (p=0.0173), Cre CER 120 days vs. Kras/Cre CER 120 days (p=0.0246), Kras/Cre CER 30 days vs. Kras/Cre Sal 30 days (p=0.0148), Cre Sal 30 days vs. Kras/Cre Sal 120 days (p=0.0269), Cre Sal 120 days vs. Kras/Cre Sal 120 days (p=0.0071), Cre Sal 120 days vs. Kras/Cre Sal 120 days (p=0.0278).
d	Cre CER 30 days vs. Kras/Cre CER 30 days (p=0.02189), Kras/Cre CER 30 days vs. Kras/Cre CER 120 days (p=0.0328), Kras/Cre CER 120 days vs. Kras/Cre Sal 120 days (p=0.0241), Cre Sal 30 days vs. Kras/Cre Sal 120 days (p=0.0353), Cre Sal 120 days vs. Kras/Cre Sal 120 days (p=0.0313).
e	Cre CER 30 days vs. Kras/Cre CER 30 days (p=0.0108), Kras/Cre CER 30 days vs. Kras/Cre Sal 30 days (p=0.0245), Cre Sal 30 days vs. Kras/Cre Sal 120 days (p=0.0010), Cre Sal 120 days vs. Kras/Cre Sal 120 days (p=0.0409), Cre Sal 30 days vs. Kras/Cre Sal 120 days (p=0.0483).
f	Cre CER 30 days vs. Kras/Cre CER 30 days (p=0.0075), Kras/Cre CER 30 days vs. Kras/Cre Sal 30 days (p=0.0187), Cre Sal 30 days vs. Kras/Cre Sal 120 days (p=0.0012), Cre Sal 120 days vs Kras/Cre Sal 120 days (p=0.0154), Cre Sal 30 days vs. Kras/Cre Sal 120 days (p=0.0149).
g	Cre CER 30 days vs. Kras/Cre CER 30 days (p=0.0283), Kras/Cre CER 30 days vs. Kras/Cre Sal 30 days (p=0.0311), Cre 30 Sal days vs. Kras/Cre Sal 120 days (p=0.0011), Cre Sal 120 days vs. Kras/Cre Sal 120 days (p=0.0026), Cre Sal 30 days vs. Kras/Cre Sal 120 days (p=0.0099).
h	Cre CER 30 days vs. Kras/Cre CER 30 days (p=0.0013), Cre CER 120 days vs. Kras/Cre CER 120 days (p=0.0056), Kras/Cre CER 30 days vs. Kras/Cre Sal 30 days (p=0.0034), Kras/Cre CER 120 days vs. Kras/Cre Sal 30 days (p=0.0144), Cre Sal 30 days vs. Kras/Cre Sal 120 days (p=0.0008), Cre Sal 120 days vs. Kras/Cre Sal 120 days (p=0.0017), Cre Sal 30 days vs. Kras/Cre Sal 120 days (p=0.0034).
i	Cre CER 30 days vs. Kras/Cre CER 30 days (p=0.0349), Kras/Cre CER 30 days vs. Kras/Cre CER 120 days (p=0.0329), Cre Sal 30 days vs. Kras/Cre Sal 120 days (p=0.0451)
j	Cre CER 30 days vs. Kras/Cre CER 30 days (p=0.0214), Cre CER 120 days vs. Kras/Cre CER 120 days (p=0.0307), Kras/Cre CER 30 days vs. Kras/Cre Sal 30 days (p=0.0177), Kras/Cre CER 120 days vs. Kras/Cre Sal 30 days (p=0.0484), Cre Sal 30 days vs. Kras/Cre Sal 120 days (p=0.0150), Cre Sal 120 days vs. Kras/Cre Sal 120 days (p=0.0152), Cre Sal 30 days vs. Kras/Cre Sal 120 days (p=0.0283).
K	Kras/Cre CER 30 days vs. Kras/Cre CER 120 days (p=0.0275), Kras/Cre CER 30 days vs. Kras/Cre Sal 30 days (p=0.0104), Cre Sal 30 days vs. Kras/Cre Sal 120 days (p=0.0230).

Kras/Cre – mice with Kras mutation, Cre mice without Kras mutation, Sal- saline, CER - cerulein

Table S8. The percentage of pancreatic samples with genera not detected in inflammation-associated pancreatic carcinogenesis.

	Cre mice				Kras/Cre mice			
	Saline		Cerulein		Saline		Cerulein	
	30 Days	120 days	30 days	120 Days	30 Days	120 days	30 Days	120 days
Prevotella	75	75	100	83,33	50	100	100	100
Lachnospiraceae A2	25	100	75	50	83,33	100	100	100
Lachnospiraceae unclassified	25	100	50	50	83,33	100	100	100
Alloprevotella	25	75	75	66,67	83,33	100	100	100
Oscillospiraceae Uncultured	25	75	75	33,33	83,33	100	100	100
Colidextribacter	25	50	50	33,33	83,33	80	100	100
Oscillibacter	25	50	75	33,33	66,67	80	100	100
Incertae Sedis	75	75	50	33,33	83,33	80	100	100

Cre mice – mice without Kras mutation, Kras/Cre – mice with Kras mutation

Table S9. The alpha diversity indexes (Shannon index and Simpson index) of pancreatic and stool microbiota mice with Kras mutation and mice without Kras mutation in inflammation-induced carcinogenesis

		**Pancreas**		**Stool**	
	**Group**	**Shannon**	**Simpson**	**Shannon**	**Simpson**
mean	Cre CER 30 days	1,141349	0,310662	4,679513	0,975862
	Cre CER 120 days	1,685565	0,42964	4,541192	0,974133
	Kras/Cre CER 30 days	1,974263	0,643433	3,014802	0,845246
	Kras/Cre CER 120 days	0,869761	0,308439	3,828764	0,913094
	Cre Sal 30 days	1,759217	0,479134	4,547697	0,972888
	Cre Sal 120 days	0,496898	0,148757	4,155115	0,951127
	Kras/Cre Sal 30 days	1,156672	0,364944	4,415094	0,971521
	Kras/Cre Sal 120 days	1,348192	0,486589	3,168529	0,864672
SD	Cre CER 30 days	1,081153	0,266953	0,240428	0,011709
	Cre CER 120 days	1,48087	0,359337	0,174495	0,006626
	Kras/Cre CER 30 days	0,869062	0,214046	0,768149	0,083447
	Kras/Cre CER 120 days	0,364587	0,150968	1,010378	0,103768
	Cre Sal 30 days	1,067675	0,272764	0,197768	0,00412
	Cre Sal 120 days	0,307234	0,104861	0,764348	0,058343
	Kras/Cre Sal 30 days	1,085568	0,340672	0,19975	0,004202
	Kras/Cre Sal 120 days	0,600988	0,225781	0,450544	0,042889
median	Cre CER 30 days	0,695343	0,203558	4,756224	0,980792
	Cre CER 120 days	1,616921	0,422524	4,496132	0,973788
	Kras/Cre CER 30 days	1,702732	0,608047	2,790617	0,8418
	Kras/Cre CER 120 days	0,842397	0,291434	3,943628	0,947346
	Cre Sal 30 days	1,381668	0,370613	4,578982	0,972076
	Cre Sal 120 days	0,439429	0,119917	4,398231	0,972036
	Kras/Cre Sal 30 days	0,541767	0,168132	4,457872	0,971115
	Kras/Cre Sal 120 days	1,471652	0,55852	3,09684	0,866099
Q1	Cre CER 30 days	0,488226	0,1467	4,434555	0,964228
	Cre CER 120 days	0,202925	0,067669	4,395225	0,969728
	Kras/Cre CER 30 days	1,341713	0,467323	2,516716	0,768488
	Kras/Cre CER 120 days	0,533655	0,174007	2,914545	0,89342
	Cre Sal 30 days	1,046719	0,307522	4,464764	0,970328
	Cre Sal 120 days	0,238394	0,068523	3,938221	0,944635
	Kras/Cre Sal 30 days	0,383252	0,13478	4,251749	0,967969
	Kras/Cre Sal 120 days	0,843693	0,307321	2,864028	0,838104
Q3	Cre CER 30 days	2,240477	0,581728	4,849268	0,984162
	Cre CER 120 days	3,204263	0,781981	4,716355	0,980562
	Kras/Cre CER 30 days	2,878344	0,854928	3,556849	0,910206
	Kras/Cre CER 120 days	1,23323	0,459876	4,856529	0,981834
	Cre Sal 30 days	2,849263	0,759266	4,672827	0,975874

	Cre Sal 120 days	0,812871	0,257832	4,60547	0,980348
	Kras/Cre Sal 30 days	2,514052	0,757586	4,557051	0,975275
	Kras/Cre Sal 120 days	1,790961	0,62989	3,351026	0,883064

Cre mice – mice without Kras mutation, Kras/Cre – mice with Kras mutation, Sal – saline, CER – cerulein, SD – standard deviation, Q1 - first quartile, Q3 – thrid quartile

Table S10. The comparison of microbiota alpha diversity indexes (Shannon index, Simpson index) in inflammation-induced carcinogenesis (p values) A. pancreas samples, B. stool samples.

A. Pancreas

		**Shannon Index**	**Simpson index**
**p-value**		2,99E-01	0,277807813
**post-hoc**	CER_Cre_30 days vs. CER_ Cre_120 days	0,3958912	0,475640909
	CER_ Cre_30 days vs. CER_Kras/Cre_30 days	0,315899882	0,291076282
	CER_ Cre_120 days vs. CER_ Kras/Cre_30 days	0,362472223	0,270574339
	CER_ Cre_30 days vs. CER_ Kras/Cre_120 days	0,464619215	0,486971758
	CER_ Cre_120 days vs. CER_ Kras/Cre_120 days	0,367299721	0,471552057
	CER_ Kras/Cre_30 days vs. CER_ Kras/Cre_120 days	0,330421679	0,265942627
	CER_ Cre_30 days vs. Sal_ Cre_30 days	0,373476538	0,406897887
	CER_ Cre_120 days vs. Sal_ Cre_30 days	0,366698801	0,363682456
	CER_ Kras/Cre_30 days vs. Sal_ Cre_30 days	0,477837048	0,378435976
	CER_ Kras/Cre_120 days vs. Sal_ Cre_30 days	0,33372479	0,369368764
	CER_ Cre_30 days vs. Sal_ Cre_120 days	0,376633854	0,321945115
	CER_ Cre_120 days vs. Sal_ Cre_120 days	0,308004875	0,323150131
	CER_ Kras/Cre_30 days vs. Sal_ Cre_120 days	0,239509689	0,103581175
	CER_ Kras/Cre_120 days vs. Sal_ Cre_120 days	0,360423995	0,335511378
	Sal_ Cre_30 days vs. Sal_ Cre_120 days	0,217726386	0,30000576
	CER_ Cre_30 days vs. Sal_ Kras/Cre_30 days	0,477181687	0,4924331
	CER_ Cre_120 days vs. Sal_ Kras/Cre_30 days	0,372066365	0,472683001
	CER_ Kras/Cre_30 days vs. Sal_ Kras/Cre 30 days	0,375806873	0,403937664
	CER_ Kras/Cre_120 days vs. Sal_ Kras/Cre_30 days	0,5	0,488937891
	Sal_ Cre_30 days vs. Sal_ Kras/Cre_30 days	0,347881293	0,390491461
	Sal_ Cre_120 days vs. Sal_ Kras/Cre_30 days	0,340757035	0,304396131
	CER_ Cre_30 days vs. Sal_ Kras/Cre_120 days	0,381178645	0,425911594
	CER_ Cre_120 days vs. Sal_ Kras/Cre_120 days	0,476417756	0,446161011
	CER_ Kras/Cre_30 days vs. Sal_ Kras/Cre_120 days	0,367888015	0,415516423
	CER_ Kras/Cre_120 days vs. Sal_ Kras/Cre_120 days	0,355499473	0,418831892
	Sal_ Cre_30 days vs. Sal_ Kras/Cre_120 days	0,364200572	0,456338924
	Sal_ Cre_120 days vs. Sal_ Kras/Cre_120 days	0,409223262	0,26770735
	Sal_ Kras/Cre_30 days vs. Sal_ Kras/Cre_120 days	0,382681898	0,398065123

Cre mice – mice without Kras mutation, Kras/Cre – mice with Kras mutation, Sal – saline,

CER - cerulein

B. Stool

		Shannon Index	Simpson Index
**p-value**		8,45E-05	9,67508E-05
**post-hoc**	CER_Cre_30 days vs. CER_Cre_120 days	0,289712145	0,331606902
	CER_ Cre_30 days vs. CER_ Kras/Cre_30 days	0,000526662	0,001055456
	CER_ Cre_120 days vs. CER_ Kras/Cre_30 days	0,004156741	0,005623108
	CER_ Cre 30 days vs. CER_ Kras/Cre_120 days	0,052039879	0,066790826
	CER_ Cre_120 days vs. CER_ Kras/Cre_120 days	0,160607606	0,234989124
	CER_ Kras/Cre_30 days vs. CER_ Kras/Cre_120 days	0,059840026	0,063511569
	CER_ Cre_30 days vs. Sal_ Cre_30 days	0,328984561	0,255060323
	CER_ Cre_120 days vs. Sal_ Cre_30 days	0,448808022	0,444922373
	CER_ Kras/Cre_30 days vs. Sal_ Cre_30 days	0,003812397	0,011075551
	CER_ Kras/Cre_120 days vs. Sal_ Cre_30 days	0,141850386	0,292320997
	CER_ Cre_30 days vs. Sal_ Cre_120 days	0,059084466	0,146944513
	CER_ Cre_120 days vs. Sal_ Cre_120 days	0,200408823	0,355539429
	CER_ Kras/Cre_30 days vs. Sal_ Cre_120 days	0,034393397	0,012094731
	CER_ Kras/Cre_120 days vs. Sal_ Cre_120 days	0,434220711	0,368954305
	Sal_ Cre_30 days vs. Sal_ Cre_120 days	0,164008312	0,429121801
	CER_ Cre_30 days vs. Sal_ Kras/Cre_30 days	0,154859938	0,252101302
	CER_ Cre_120 days vs. Sal_ Kras/Cre_30 days	0,322125032	0,419586629
	CER_ Kras/Cre_30 days vs. Sal_ Kras/Cre_30 days	0,052604628	0,033211439
	CER_ Kras/Cre_120 days vs. Sal_ Kras/Cre_30 days	0,383620235	0,346772972
	Sal_ Cre_30 days vs. Sal_ Kras/Cre_30 days	0,291248178	0,44798029
	Sal_ Cre_120 days vs. Sal_ Kras/Cre_30 days	0,440517059	0,457782591
	CER_ Cre_30 days vs. Sal_ Kras/Cre_120 days	0,001052759	0,000510176
	CER_ Cre_120 days vs. Sal_ Kras/Cre_120 days	0,003742892	0,003748033
	CER_ Kras/Cre_30 days vs. Sal_ Kras/Cre_120 days	0,454360519	0,440802274
	CER_ Kras/Cre_120 days vs. Sal_ Kras/Cre_120 days	0,063556787	0,037956835
	Sal_ Cre_30 days vs. Sal_ Kras/Cre_120 days	0,00304647	0,00715578
	Sal_ Cre_120 days vs. Sal_ Kras/Cre_120 days	0,033768626	0,006438054
	Sal_Kras/Cre_30 days vs. Sal_ Kras/Cre_120 days	0,053935957	0,018712066

Cre mice – mice without Kras mutation, Kras/Cre – mice with Kras mutation, Sal – saline, CER - cerulein

Table S11. The significant differences (p value) for comparison in the alpha diversity index in Figure 3.

Figure 3	P value
b	Cre CER 30 days vs. Kras/Cre CER 30 days (p=0.0005), Cre CER 120 days vs. Kras/Cre CER 30 days (p=0.0042), Kras/Cre CER 30 days vs. Cre Sal 30 days (p=0.0038), Kras/Cre CER 30 days vs. Cre Sal 120 days (p=0.0344), Cre CER 30 days vs. Kras/Cre Sal 120 days (p=0.0011), Cre CER 120 days vs. Kras/Cre Sal 120 days (p=0.0037), Cre Sal 30 days vs. Kras/Cre Sal 120 days (p=0.0030), Cre Sal 120 days vs. Kras/Cre Sal 120 days (p=0.0338).

Kras/Cre – mice with Kras mutation, Cre mice without Kras mutation, CER – cerulein, Sal - saline

Table S12. The prevalence and abundance of phyla in pancreas samples between Cre mice and Kras/Cre mice after fecal microbiota transplantation and sham treatment.

Cre mice – mice without Kras mutation, Kras/Cre – mice with Kras mutation, FMT – fecal microbiota transplantation

Relative abundance: pancreas phyla											
% reads											
Group	Actinobacter	Bacteroid	Campilobacter	Cyanobac	Deferribacte	Desulfobacte	Firmicut	Patescibac	Proteobact	Spirochae	Verrucomicrobiota
SHAM_Cre	18,1203988	14,5905	12,42125668	4,09286	0	1,55861519	20,47	0	24,93928	0	3,8075
SHAM_Kras/Cre	17,8772498	13,5293	12,72731467	0	4,3784238	6,92344535	17,681	3,009359	23,02413	0	0,84973
FMT_Cre	14,8744107	12,3078	13,75566545	0,60248	3,7563468	6,27829875	18,648	1,175696	23,46157	1,34129	3,79885
FMT_Kras/Cre	19,359727	12,8011	13,28656502	2,38256	4,9942756	2,79082743	17,095	2,455027	22,56674	0,74773	1,52045

Prevalance: pancreas phyla											
%											
Group	Actinobacter	Bacteroid	Campilobacter	Cyanobac	Deferribacte	Desulfobacte	Firmicut	Patescibac	Proteobact	Spirochae	Verrucomicrobiota
SHAM_Cre	100	100	80	40	0	20	100	0	100	0	40
SHAM_Kras/Cre	100	100	100	0	60	80	100	40	100	0	20
FMT_Cre	100	85,7143	100	14,2857	57,142857	85,7142857	100	14,28571	100	14,2857	57,1429
FMT_Kras/Cre	100	100	100	33,3333	66,666667	66,6666667	100	44,44444	100	11,1111	33,3333

Table S13. The prevalence and abundance of phyla in stool samples between Cre mice and Kras/Cre mice after fecal microbiota transplantation and sham treatment.

Cre mice – mice without Kras mutation, Kras/Cre – mice with Kras mutation, FMT – fecal microbiota transplantation

Relative abundance: stool phyla											
% reads											
Group	Actinobacte	Bacteroid	Campilobacte	Cyanoba	Deferribacte	Desulfobacte	Firmicut	Patescibac	Proteobact	Spirocha	Verrucomicrobiota
SHAM_Cre	12,185156	16,51669	14,60761061	0	0	12,38014257	17,933	9,931177	7,5250032	0	8,921457
SHAM_Kras/Cre	14,8208389	14,85297	13,84308035	3,39669	8,23638925	6,826268118	16,416	5,805338	8,2813385	0	7,52096
FMT_Cre	9,2188465	13,54563	11,56436815	0,67868	7,80507451	10,06322706	14,771	8,578705	7,9485341	7,78383	8,042205
FMT_Kras/Cre	12,8943662	13,40901	11,87071526	3,14122	8,58846098	8,014854472	14,264	6,77078	8,9073618	5,61607	6,522902

Prevalance: stool phyla											
%											
Group	Actinobacte	Bacteroid	Campilobacte	Cyanoba	Deferribacte	Desulfobacte	Firmicut	Patescibac	Proteobact	Spirocha	Verrucomicrobiota
SHAM_Cre	100	100	100	0	0	100	100	100	100	0	100
SHAM_Kras/Cre	100	100	100	66,6667	83,3333333	83,33333333	100	66,66667	100	0	100
FMT_Cre	100	100	100	50	100	83,33333333	100	83,33333	100	16,6667	100
FMT_Kras/Cre	100	100	100	75	87,5	100	100	87,5	100	87,5	100

.

Table S14. The significant differences (p value) for comparison in the phyla abundances presented in Figure 4.

Figure 4	P value
b	Cre FMT vs. Kras/Cre FMT (p=0.0017), Kras/Cre FMT vs. Cre SHAM (p=0.0080).
c	Cre FMT vs. Kras/Cre FMT (p=0.0013), Kras/Cre FMT vs. Cre SHAM (p=0.0158), Cre FMT vs. Kras/Cre SHAM (p=0.0014), Cre SHAM vs. Kras/Cre SHAM (p=0.0154)
d	Cre FMT vs. Kras/Cre FMT (p=0.0419), Cre FMT vs. Kras/Cre SHAM (p=0.0458), Cre SHAM vs. Kras/Cre SHAM (p=0.0437)
e	Cre FMT vs. Cre SHAM (p=0.0311), Kras/Cre FMT vs. Cre SHAM (p=0.0020), Cre SHAM vs. Kras/Cre SHAM (p=0.0230)
f	Cre FMT vs. Kras/Cre FMT (p=0.0272), Cre FMT vs. Kras/Cre SHAM (p=0.0106), Cre SHAM vs. Kras/Cre SHAM (p=0.0356)
g	Cre FMT vs. Cre SHAM (p=0.0401), Kras/Cre FMT vs Cre SHAM (p=0.0012), Kras/Cre FMT vs. Kras/Cre SHAM (p=0.0485)
h	Cre FMT vs Kras/Cre FMT (p=0.0466), Cre FMT vs. Cre SHAM (p=0.0003), Kras/Cre FMT vs. Cre SHAM (p=0.0234), Cre FMT vs. Kras/Cre SHAM (p=0.0003), Kras/Cre FMT vs. Kras/Cre SHAM (p=0.0229).

Kras/Cre – mice with Kras mutation, Cre mice without Kras mutation, FMT – fecal microbiota transplantation, SHAM – sham treatment

Table S15. The prevalence and abundance of the most common bacterial genera in pancreas samples in the fecal microbiota transplantation associated pancreatic carcinogenesis.

Cre mice – mice without Kras mutation, Kras/Cre – mice with Kras mutation, FMT – fecal microbiota transplantation

Relative abundance: pancreas genera										
% reads										
Group	Escherichi	Bifidobact	Lactobacil	Dubosiella	Bacteroide	Lachnospi	Faecalibac	Muribacul	Lachnospi	Alistipes
SHAM_Cre	21,51524	15,65135	16,51451	4,865411	9,148012	7,817182	1,447358	10,71531	7,764503	4,561119
SHAM_Kras/Cre	19,61549	14,74029	11,78966	10,18862	8,791557	8,390928	4,761562	8,217161	8,384268	5,120466
FMT_Cre	21,43141	13,3118	12,44692	5,975949	7,229855	12,0093	1,001664	8,798083	9,28008	8,514941
FMT_Kras/Cre	18,39712	15,7268	11,99465	10,56722	8,920491	8,265391	7,33579	6,922749	6,695203	5,17458

Prevalance: pancreas genera										
%										
Group	Escherichi	Bifidobact	Lactobacil	Dubosiella	Bacteroide	Lachnospi	Faecalibac	Muribacul	Lachnospi	Alistipes
SHAM_Cre	100	100	100	60	100	80	20	100	60	40
SHAM_Kras/Cre	100	100	100	100	100	80	60	80	80	60
FMT_Cre	100	100	85,71429	57,14286	71,42857	100	14,28571	85,71429	85,71429	85,71429
FMT_Kras/Cre	100	100	100	100	100	100	100	85,71429	100	85,71429

Table S16. The prevalence and abundance of the most common bacterial genera in stool samples in the fecal microbiota transplantation associated pancreatic carcinogenesis.

Cre mice – mice without Kras mutation, Kras/Cre – mice with Kras mutation, FMT – fecal microbiota transplantation

Relative abundance: stool genera

% reads

Group	Bifidobact	Muribacul	Lactobacil	Lachnospi	Bacteroide	Mycoplasr	Mucispirill	Alistipes	Lachnospi	Faecalibaculum
SHAM_Cre	10,61253	13,70752	11,78219	12,99749	11,06319	12,49164	0	11,77981	12,95658	2,609062
SHAM_Kras/Cre	13,25059	11,65211	13,22965	11,19834	11,8489	9,096494	7,400421	8,117199	7,4826	6,723698
FMT_Cre	8,0463	12,49146	11,67622	12,22387	10,24549	12,34932	7,697963	11,00124	11,61713	2,651006
FMT_Kras/Cre	12,6945	12,40703	12,33233	10,54974	10,40238	9,265362	8,474004	8,157569	7,992768	7,724322

Prevalance: stool genera

%

Group	Bifidobact	Muribacul	Lactobacil	Lachnospi	Bacteroide	Mycoplasr	Mucispirill	Alistipes	Lachnospi	Faecalibaculum
SHAM_Cre	100	100	100	100	100	100	0	100	100	60
SHAM_Kras/Cre	100	100	100	100	100	83,33333	83,33333	100	66,66667	100
FMT_Cre	85,71429	100	100	100	100	100	100	100	100	57,14286
FMT_Kras/Cre	100	100	100	100	100	100	87,5	100	87,5	100

Table S17. The significant differences (p value) for comparison in the genera abundances presented in Figure 5.

Figure 5	P value
b	Cre FMT vs Kras/Cre FMT (p=0.0020), Kras/Cre FMT vs. Cre SHAM (p=0.0083)
c	Cre FMT vs Kras/Cre FMT (p=0.0183), Kras/Cre FMT vs. Cre SHAM (p=0.0054)
d	Cre FMT vs Kras/Cre FMT (p=0.0089), Kras/Cre FMT vs. Cre SHAM (p=0.0149)
e	Cre FMT vs Kras/Cre FMT (p=0.0132)
f	Cre FMT vs Kras/Cre FMT (p=0.0455)
g	Cre FMT vs Kras/Cre FMT (p=0.0090), Cre FMT vs. Cre SHAM (p=0.0269)
h	Cre FMT vs Kras/Cre FMT (p=0.0496), Cre FMT vs. Cre SHAM (p=0.0312)
i	Cre FMT vs Kras/Cre FMT (p=0.0467), Cre FMT vs. Cre SHAM (p=0.0415)

Kras/Cre – mice with Kras mutation, Cre mice without Kras mutation, FMT – fecal microbiota transplantation, SHAM – sham treatment

Table S18. The differences in genera abundance in pancreas samples between Cre mice and Kras/Cre mice after fecal microbiota transplantation and sham treatment.

Excel file Suppl. Table 18

Cre mice – mice without Kras mutation, Kras/Cre – mice with Kras mutation, FMT – fecal microbiota transplantation

Table S19. The differences in genera abundance in stool samples between Cre mice and Kras/Cre mice after fecal microbiota transplantation and sham treatment.

Excel file Supp. Tabl. 19

Cre mice – mice without Kras mutation, Kras/Cre – mice with Kras mutation, FMT – fecal microbiota transplantation

Analyzing these changes after sham and FMT treatments, we observed five main trends in microbial composition.

First, there were several genera that did not change in Cre mice after both FMT and sham treatments, but were changed in Kras/Cre mice after both treatments e.g. *Bifidobacterium, Faecalibaculum, Roseburia, Desulfovibrionaceae uncultured, Lachnospiraceae A2, Lachnospiraceae ASF356, Coriobacteriaceae UCG-002, Erysipelotrichaceae uncultured, Clostridia vadinBB60 group, Lachnospiraceae Blautia, Lachnospiraceae GCA-900066575, Eubacterium xylanophilum group, Butyricicoccus, Oscillospiraceae NK4A214 group, Oscillibacter, Oscillospiraceae uncultured, Incertae Sedis*, and *Negativibacillus*.

Secondly, the FMT in Kras-Cre mice only compared to Cre resulted in significant change in the abundance of *Alistipes, Rikinella, Mycoplasma, Lachnoclostridium, Lachnospiraceae FCS020 group, Marvinbryantia, Peptococcaceae uncultured,* and *Puniceicoccaceae uncultured.*

Third, FMT, compared to the sham treatment in both Cre mice and Kras/Cre mice significantly changed the abundance of *Alloprevotella, Desulfovibrio, Parasutterella, and Brachyspira*.

In addition, there were significant differences in the abundance of *Rikenellaceae RC9 gut group* and *Ruminococcaceae* between Kras/Cre mice and Cre mice after FMT and between Cre mice after FMT and sham treatment.

Finally, there were only two genera such as *Odoribacter and Prevotellaceae UCG-001*, that significantly changed in Kras/Cre mice after FMT compared to the sham treatment, but also were different between Kras/Cre mice after sham treatment compared to Cre mice after sham treatment.

Table S20. The significant differences (p value) for comparison in the genera abundances presented in Figure 6.

Figure 6	P value
a	Cre FMT vs. Kras/Cre FMT (p=0.0024), Kras/Cre FMT vs. Cre SHAM (p=0.0122), Cre FMT vs. Kras/Cre SHAM (p=0.0023), Cre SHAM vs. Kras/Cre SHAM (p=0.0122)
b	Cre FMT vs. Kras/Cre FMT (p=0.0092), Kras/Cre FMT vs. Cre SHAM (p=0.0072), Cre FMT vs. Kras/Cre SHAM (p=0.0468), Cre SHAM vs. Kras/Cre SHAM (p=0.0386)
c	Cre FMT vs. Kras/Cre FMT (p=0.0094), Kras/Cre FMT vs. Cre SHAM (p=0.0055), Cre FMT vs. Kras/Cre SHAM (p=0.0076), Cre SHAM vs. Kras/Cre SHAM (p=0.0101
d	Cre FMT vs. Kras/Cre FMT (p=0.0252), Kras/Cre FMT vs. Cre SHAM (p=0.0364), Cre FMT vs. Kras/Cre SHAM (p=0.0256), Cre SHAM vs. Kras/Cre SHAM (p=0.0273)
e	Cre FMT vs. Kras/Cre FMT (p=0.0144), Kras/Cre FMT vs. Cre SHAM (p=0.0139), Cre FMT vs. Kras/Cre SHAM (p=0.0147), Cre SHAM vs. Kras/Cre SHAM (p=0.0170)
f	Cre FMT vs. Kras/Cre FMT (p=0.0158), Kras/Cre FMT vs. Cre SHAM (p=0.0315), Cre FMT vs. Kras/Cre SHAM (p=0.0065), Cre SHAM vs. Kras/Cre SHAM (p=0.0109)

Kras/Cre – mice with Kras mutation, Cre mice without Kras mutation, FMT – fecal microbiota transplantation, SHAM - sham treatment (control)

Table S21. The diversity index (Shannon index) in Kras/Cre mice and Cre mice after fecal microbiota transplantation.

	Group	Shannon index	
		Pancreas	Stool
average	Cre Sham	1,772661	4,471194
	Kras/Cre Sham	1,328841	3,361221
	Cre FMT	1,387141	4,273516
	Kras/Cre FMT	1,243549	3,221184
SD	Cre Sham	1,491417	0,149754
	Kras/Cre Sham	0,457432	1,033189
	Cre FMT	0,785694	0,455577
	Kras/Cre FMT	0,363809	0,735584
median	Cre Sham	1,619991	4,488205
	Kras/Cre Sham	1,475267	3,536597
	Cre FMT	1,338069	4,445858
	Kras/Cre FMT	1,366864	3,269545
Q1	Cre Sham	0,334701	4,353331
	Kras/Cre Sham	0,891725	2,254997
	Cre FMT	0,82663	4,084009
	Kras/Cre FMT	0,830566	2,719058
Q3	Cre Sham	3,286956	4,580551
	Kras/Cre Sham	1,692744	4,303944
	Cre FMT	1,696172	4,533466
	Kras/Cre FMT	1,507757	3,563492

Cre mice – mice without Kras mutation, Kras/Cre – mice with Kras mutation, FMT – fecal microbiota transplantation, SD- standard deviation, Q1 – first quartile, Q3 – third quartile

Table S22. The significant differences (p value) for comparison in the alpha diversity index in Figure 7.

Figure 7	P value
b	Cre FMT vs. Kras/FMT (p=0.0223), Kras/Cre FMT vs. Cre SHAM (p=0.0251), Cre FMT vs. Kras/Cre SHAM (p=0.0288), Cre SHAM vs. Kras/Cre SHAM (p=0.0213)

Kras/Cre – mice with Kras mutation, Cre mice without Kras mutation, FMT – fecal microbiota transplantation, SHAM – sham treatment
